# The transient Spt4-Spt5 complex as an upstream regulator of non-coding RNAs during development

**DOI:** 10.1093/nar/gkac106

**Published:** 2022-02-21

**Authors:** Dawid Owsian, Julita Gruchota, Olivier Arnaiz, Jacek K Nowak

**Affiliations:** Institute of Biochemistry and Biophysics, Polish Academy of Sciences, Pawinskiego 5a, 02-106 Warsaw, Poland; Institute of Biochemistry and Biophysics, Polish Academy of Sciences, Pawinskiego 5a, 02-106 Warsaw, Poland; Université Paris-Saclay, CEA, CNRS, Institute for Integrative Biology of the Cell (I2BC), 91198, Gif-sur-Yvette, France; Institute of Biochemistry and Biophysics, Polish Academy of Sciences, Pawinskiego 5a, 02-106 Warsaw, Poland

## Abstract

The Spt4-Spt5 complex is conserved and essential RNA polymerase elongation factor. To investigate the role of the Spt4-Spt5 complex in non-coding transcription during development, we used the unicellular model *Paramecium tetraurelia*. In this organism harboring both germline and somatic nuclei, massive transcription of the entire germline genome takes place during meiosis. This phenomenon starts a series of events mediated by different classes of non-coding RNAs that control developmentally programmed DNA elimination. We focused our study on Spt4, a small zinc-finger protein encoded in *P. tetraurelia* by two genes expressed constitutively and two genes expressed during meiosis. *SPT4* genes are not essential in vegetative growth, but they are indispensable for sexual reproduction, even though genes from both expression families show functional redundancy. Silencing of the *SPT4* genes resulted in the absence of double-stranded ncRNAs and reduced levels of scnRNAs – 25 nt-long sRNAs produced from these double-stranded precursors in the germline nucleus. Moreover, we observed that the presence of a germline-specific Spt4-Spt5m complex is necessary for transfer of the scnRNA-binding PIWI protein between the germline and somatic nucleus. Our study establishes that Spt4, together with Spt5m, is essential for expression of the germline genome and necessary for developmental genome rearrangements.

## INTRODUCTION

The Spt4/Spt5 complex is a conserved transcription elongation factor present in eukaryotes and Archaea (1). It affects the processivity of RNA polymerase II in both a repressive and stimulatory manner, standing as an unique regulator of transcription. The complex attaches to the polymerase during the early phase of elongation, stimulating its stoppage at the promoter proximal region. Due to the activity of specific kinases, both polymerase II and the Spt4/Spt5 heterodimer are phosphorylated which causes transition of the holoenzyme into the state of productive elongation. Finally, during the last stage of transcription, the Spt4/Spt5 complex is dephosphorylated which decelerates the ongoing polymerase II and facilitates termination of the whole process (2–5).

According to genetic analysis both proteins forming the heterodimer play important roles in enzyme distribution and transcriptional output (2,6–8). Nonetheless their mutual relation remains elusive and some results lead to ambiguous conclusions. For instance Spt5 as a multidomain protein providing physical interactions with polymerase II is essential for vegetative growth (9,10). On the contrary, Spt4, which is a small protein consisting of a zinc-binding motif and a domain interfacing with Spt5, is not required for asexual reproduction (11). In addition, in some biological processes they function independently of each other. As an example, Spt5 is involved in RNA maturation, whereas Spt4 is involved in chromosome transmission and the assembly of centromere heterochromatin (12–15). Furthermore Spt4 functions as a negative regulator of autophagy through inhibition of Spt5 phosphorylation, suggesting that their roles do not necessarily overlap (16).

Considering the results of known whole-genome analyses, the regulation of gene expression and the biogenesis of small regulatory transcripts during development involve stalling of polymerase II which at least partially depends upon the activity of the Spt4/Spt5 complex (17–19). Consistently, mutation of the Spt5 homologue in zebrafish impairs neurodevelopment whilst its depletion in *P. tetraurelia* disturbs the synthesis of small non-coding RNAs, leading to death of the post-sexual progeny (20,21). In addition, a knockout of Spt4 causes embryonic lethality in mice (22). Taken together, the available data show that both proteins are engaged in developmental processes, but further research is needed to help us understand the biological significance of their activity.

To study the mechanisms that govern the processes preceding fertilization, an appropriate model is required. *Paramecium tetraurelia* is an eukaryotic single cell organism, containing a polyploid somatic macronucleus (MAC) and two diploid germline micronuclei (MICs). Nuclear dimorphism provides a unique opportunity to observe gametogenesis, karyogamy and non-coding RNAs-dependent genome rearrangements within a single cell. At the initial stage of the *Paramecium* sexual cycle, global transcription of the germline genome occurs. Nascent transcripts are then processed by Dicer-like proteins, giving rise to duplexes of short non-coding scnRNAs. Subsequently, those small RNAs are loaded onto Piwi-like proteins and transmitted into the maternal somatic nucleus. The introduced RNAs interact with the maternal transcriptome causing selection of those scnRNAs that are not homologous to somatic sequences. During the final stage of the cycle, epigenetically selected small RNAs are transferred to newly developing MACs, leading to mark germline-specific DNA sequences for elimination (23).

During formation of the new macronuclei nearly 30% of the total genome sequence is eliminated by the domesticated transposase PiggyMac (Pgm). Repetitive sequences of transposons and minisatelites are excised in an imprecise manner resulting in chromosome fragmentation, whereas ∼45 000 unique Internal Eliminated Sequences (IESs) are removed precisely (24). Although the mechanism of DNA excision needs further evaluation, it has been demonstrated by other authors that it is coordinated by another class of short non-coding transcripts (iesRNAs) and by the histone posttranslational modifier Ezl1 (25,26).

A previous analysis revealed that the *Paramecium* genome contains two Spt5 homologs and that they are characterized by distinct patterns of expression and localization during the cell cycle. Moreover, analysis of the meiosis-specific Spt5m protein showed its relevance in the proper execution of sexual reproduction (21). In the current study we present a functional analysis of Spt4 proteins, demonstrating their role in the biogenesis of small non-coding RNAs and also their relations with Spt5 as well as with proteins involved in the RNAi pathway.

## MATERIALS AND METHODS

### Reagents

#### Indirect immunofluorescence

In case of transgenes fused with GFP, *Paramecia* were fixed 10 min with 1% PAF in 1xPHEM, washed in TBST, soaked in TBST containing DAPI for 10 min and mounted on slides using Vectashield (Vector Laboratories). 3xFLAG carrying clones, after fixation, were permeabilized for 4 min with 1% Triton X-100 in 1xPHEM, then washed 2 times in TBST and incubated for 1 h with 1:500 anti-FLAG primary antibody (M2, Merck, F1804), washed 2 times in TBST, incubated 30 min with 1:500 goat anti-mouse Alexa488 secondary antibody (Thermo Fisher. A28175), washed 2 times in TBST and once with TBST containing DAPI. Double stranded RNAs were visualized using 1:100 overnight incubation with mouse anti-dsRNA antibody (J2, SCICONS). Cells were prepared according to the procedure developed for histone modification in (25).

#### sRNAs sequencing

RNA was extracted using the RNAzol RT reagent (MRC) from cells subjected to silencing conditions (*SPT4*-RNAi, *SPT4m*-RNAi and control) collected at different stages of the sexual cycle. The obtained small RNA samples (<200 nt) were resolved by PAGE electrophoresis with subsequent staining with SYBR Gold Nucleic Acid Gel Stain (Thermo Fisher Scientific) and excision of 20–30 nt sRNA fractions. Sequencing and analysis of sRNAs were carried out as previously described (21). Individual sequencing data sets are listed in the Supplementary Table S1.

#### mRNA sequencing

mRNA fraction was enriched using NEBNext Poly(A) mRNA Magnetic Isolation Module (New England Biolabs). Sequencing libraries were prepared using KAPA RNA HyperPrep (KAPA Biosystems) and adapters IDT for Illumina TruSeq DNA UD Indexes (Illumina) from 1 μg of material by 10 rounds of amplification. Sequencing was performed by Illumina NovaSeq 6000 with NovaSeq 6000 S1 Reagent Kit v. 1.5 (200 cycles) (Illumina) using standard procedure and addition of 0,5% Phix control library (Illumina).

#### Protein isolation and immunobloting

Cell lines transformed with constructs carrying the 3xFLAG tag fused to the genes of interest were cultured in 1xWGP. Cells were collected during the initial stage of the sexual cycle (30% meiosis and 20% fragmented MAC). 200 ml of ∼4000 cells/ml culture was spun, washed in 10 mM Tris pH 7.4 and quick-frozen in a final volume of 200 μl. Subsequently, 3 volumes of lysis buffer (350 mM NaCl, 50 mM Tris–HCl pH 7.4, 5 mM MgCl2, 1 mM DTT, 1 mM PMSF, protease inhibitor EDTA-free (Pierce)) were added to each sample and samples were thawed on ice by careful up and down pipetting. For complete lysis, cells were subjected to the freeze/thaw procedure three more times and the obtained whole cell extracts were centrifuged at 14 000 rpm for 30 min, 4ºC. In the next step supernatants were incubated with 30 μl of anti-FLAG M2 agarose resin (Merck) for 2 h at 4ºC. The washing step was conducted in the following order: one wash using 1 ml of lysis buffer, two washes with 1 ml of washing buffer containing 0.2% NP-40, 350 mM NaCl, 50 mM Tris–HCl pH 7.4 and two washes with 1 ml of 150 mM NaCl, 50 mM Tris pH 7.4. Elution was performed with 100 μl of 0.1 M glycine or with a 3xFLAG competitive peptide. Samples from the obtained eluates were mixed with 4x Laemmli Sample Buffer (Bio-Rad), denaturated 15 min at 70ºC and subjected to SDS-PAGE. Subsequently proteins were transferred to Immun-Blot PVDF membrane (Bio-Rad) and blocked in TBST with 5% (w/v) skim milk. Next, blots were incubated 1 h at RT with the primary antibody (anti-FLAG M2) diluted 1:4000 in blocking solution. The blot was washed 3 times in TBST (0.1% Tween 20) and incubated 45 min with the secondary antibody (anti-mouse HRP, Promega) diluted 1:8000 in TBST. Finally, blots were visualized using a chemiluminescence kit (Advansta) and the Alliance Q9 UVITEC Cambridge imaging system.

#### Mass spectrometry

Eluates validated by immunobloting analysis were subjected to the mass spectrometry analysis performed at the Mass Spectrometry Facility (IBB PAS, Warsaw, Poland). The standard procedure of trypsin digestion was performed, during which proteins were reduced with 0.5 M (5 mM f.c.) TCEP for 1 h at 60°C, blocked with 200 mM MMTS (10 mM f.c.) for 10 min at RT and digested overnight with 10 ng/ul trypsin. The resulting peptide mixtures were applied to a RP-18 pre-column (Waters, Milford, MA) using water containing 0.1% FA as mobile phase and then transferred to a nano-HPLC RP-18 column (internal diameter 75 μM, Waters, Milford MA) using an ACN gradient (0–35% ACN in 160 min) in the presence of 0.1% FA at a flow rate of 250 nl/min. The column outlet was coupled directly to the ion source of a Q Exactive mass spectrometer (Thermo Electron Corp., San Jose, CA) working in the regime of data-dependent MS to MS/MS switch. A blank run ensuring the absence of cross-contamination from previous samples preceded each analysis.

### Biological resources

#### Constructs

The plasmids pCSPT4mB-GFP and pCSPT4vA-GFP encoding the *SPT4mB* and *SPT4vA* genes fused at the C termini with GFP (preceded by a flexible linker) as well as the plasmids pCSPT4mB_3xFLAG, pCSPT5m_3xFLAG and pCRPB2k_3xFLAG encoding *SPT4mB*, *SPT5m* and *RPB2* fused directly with 3xFLAG sequences inserted before their stop codons, were obtained by cloning into the pCRscript vector (Invitrogen) by the overlapping PCR method (27) using SLIC (sequence and ligation independent cloning). All constructs contain putative promoter regions, open reading frames and putative terminators (genomic coordinates of the cloned fragments: *SPT4mB* – 31400..31789 of the acc. no. CAAL01001588.1; *SPT4vA* – 17205..17741 of the acc. no. CAAL01000597.1; *SPT5m* – 164290..166209 of the acc. no. CAAL01001700; *RPB2* - 4952..9062 of the acc. no. CAAL01000864).

#### GFP/FLAG transgene localization

Linearized plasmids carrying transgenes were microinjected into the MAC of vegetative 51 *nd7-1* cells, as described previously (28). Transformants were selected on the basis of nd7-1 phenotype reversal. Trichocyst discharging clones were grown in standard 0.25xWGP containing *K. pneumoniae* or in silencing medium. 10–15 ml of the cultures was harvested and used for observations of vegetative cells and progression of the sexual process from meiosis to the formation of new macronuclei. All microscope observations and imaging were performed at the Fluorescence Microscopy Facility IBB PAS.

#### Gene silencing

RNAi was performed as previously (29) using the *Paramecium tetraurelia* strain 51new (30). Plasmids used for RNAi experiments were obtained by cloning of the coding sequences of *SPT4* genes (genomic coordinates: *SPT4mA*- 185113….185421 of the acc. no. CAAL01000070.1; *SPT4mB* – 31454….31762 of the acc. no. CAAL01001588.1; *SPT4vA* – 17354….17665 of the acc. no. CAAL01000597.1; *SPT4vC* – 32966….33273 of the acc. no. CAAL01001546.1) between T7 promoters of the vector L4440 (31). As the *SPT4mA* and *SPT4mB* genes share two stretches of identical nucleotides (32 and 36 nt), it is possible that cross-silencing between these two genes takes place. As a control we used silencing medium containing induced *Escherichia coli* harboring *ND7*- or *ICL7a*-silencing plasmids (p0ND7c (32) and pICL7a (33), respectively), which target non-essential genes, or standard *Klebsiella pneumoniae* medium. For silencing of *SPT5m* and *DCL2/DCL3* we used a plasmid described previously (21,34). Procedures for the evaluation of RNAi phenotypes during vegetative growth as well as survival after the sexual cycle were conducted as described in (21).

#### IES retention

Cells cultured upon silencing of *SPT4* genes (*SPT4* RNAi, *SPT4m* RNAi) were collected at the closing stage of the sexual cycle. Afterwards, genomic DNA isolated from an enriched fraction of developing MACs (obtained as previously (24)) was subjected to Illumina pair-end sequencing. Sequencing data were mapped on the MAC (ptetraurelia_mac_51.fa), MAC + IES (ptetraurelia_mac_51_with_ies.fa) and MIC (ptetraurelia_mic2.fa) reference genomes (24,35) using Bowtie2 (v2.2.3 –local). ParTIES (v1.2 default parameters) software was used to calculate the IES retention score (36).

#### TE and MIC genome coverage

The DNA and RNA TE coverage were evaluated on bowtie2 (v2.2.3 default parameters) alignments on TE consensuses (38 LINEs, 12 TIRs, 7 Solo-ORF and 4 SINEs) (35). The number of reads (samtools v1.9) mapping on each consensus were normalized by the TE length and the number of mapped reads on MIC genome (RPKM). The heatmaps were generated with ComplexHeatmap R package and ordered by hierarchical clustering. The MIC genome coverage was evaluated using a 1-kb windows normalized coverage strategy previously described in (35,37).

### Computational resources

BLAST search and analysis of off-target siRNA matches were performed using ParameciumDB resources at https://paramecium.i2bc.paris-saclay.fr/ website (38). Protein sequences were aligned using Clustal Omega at https://www.ebi.ac.uk website (39). Phylogentic tree was computed using a PhyML phylogeny at http://phylogeny.fr website (40).

### Statistical analyses

#### Analysis of mass spectrometry data

The acquired MS/MS data were preprocessed with Mascot Distiller software (v. 2.5/2.6, MatrixScience, London, UK) and a search was performed with the Mascot Search Engine (MatrixScience, London, UK, Mascot Server 2.5/2.6) against the *Paramecium* protein (40 460 sequences; 17901632 residues) database. To reduce mass errors, the peptide and fragment mass tolerance settings were established separately for individual LC–MS/MS runs after a measured mass recalibration, as described previously (41). The Mascot search parameters were as follows: mass tolerance for peptides: typically 5 ppm, mass tolerance for fragments: typically 0.01 Da, enzyme, Trypsin; missed cleavages, 1; fixed modifications, Methylthio (C); variable modifications, Oxidation (M); instrument, HCD. The FDR was calculated and results were filtered to acquire 1% false positive identifications. Details of all mass spectrometry experiments are listed in Supplementary Table S2.

## RESULTS

### Two Spt4 families in *Paramecium*

**Figure 1.**
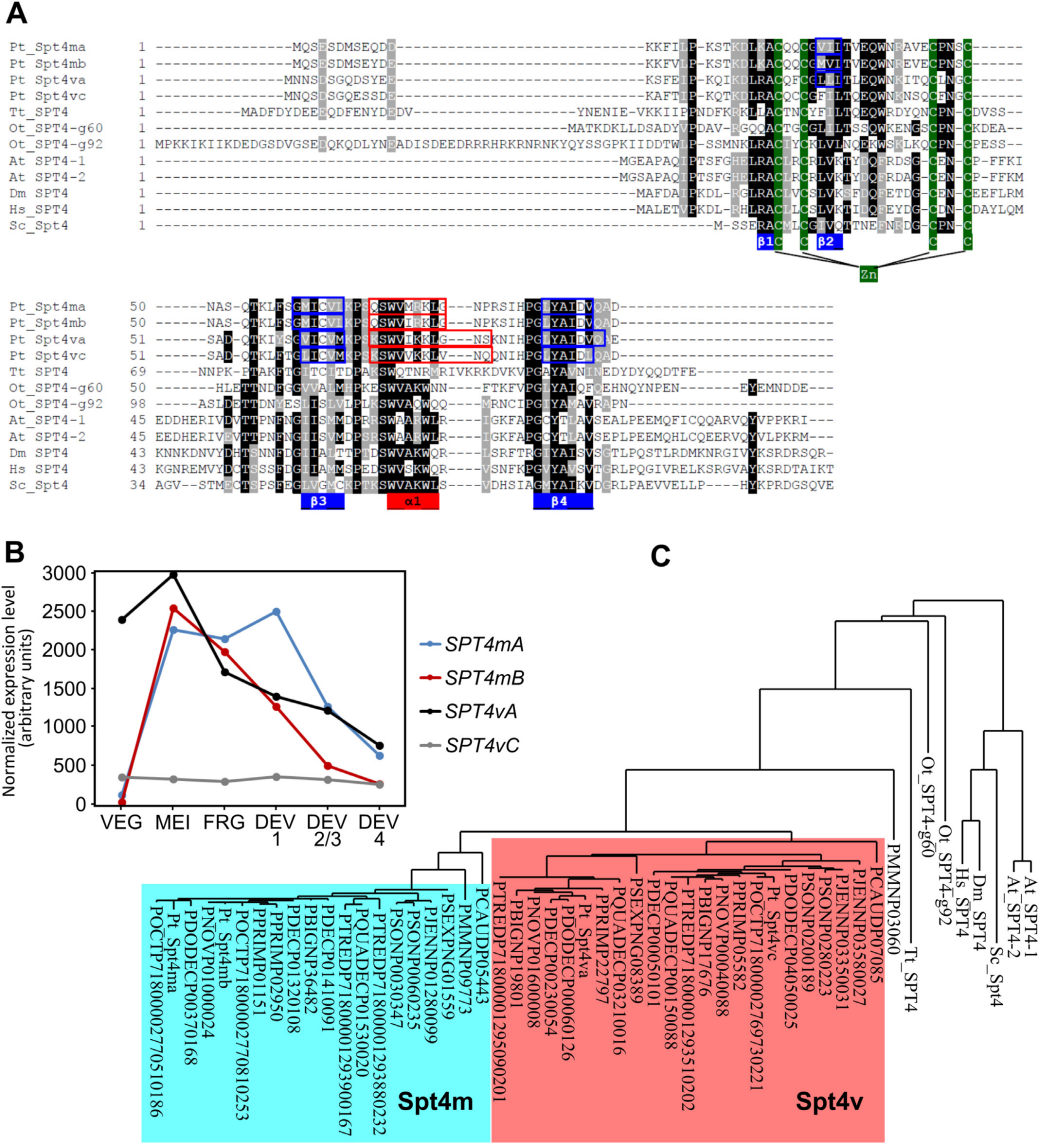
Spt4 proteins in *Paramecium*. (**A**) Alignment of Spt4 amino acid sequences from *Paramecium tetraurelia – Pt, Tetrahymena thermophila – Tt, Oxytricha trifallax – Ot, Arabidopsis thaliana – At, Drosophila melanogaster – Dm, Homo sapiens – Hs, Saccharomyces cerevisiae – Sc*. (**B**) Normalized expression level (arbitrary units) of *SPT4* genes from *Paramecium tetraurelia* during vegetative cell growth and throughout the sexual process. (**C**) Approximate likelihood tree of Spt4 proteins from ciliates and other eukaryotes based on the Clustal O alignment of entire protein sequences and on a PhyML phylogeny performed using Phylogeny.fr (40). Proteins forming the Spt4m and Spt4v subgroups in Paramecium are marked by color boxes. *Paramecium primaurelia* – PPRIM, *Paramecium biaurelia* – PBI, *Paramecium sexaurelia* – PSEX, *Paramecium caudatum* – PCAUD, *Paramecium multimicronucleatum* – PMMN, *Paramecium decaurelia* – PDEC, *Paramecium dodecaurelia* – PDODEC, *Paramecium jenningsi* – PJEN, *Paramecium novaurelia* – PNOV, *Paramecium octaurelia* – POCT, *Paramecium quadecaurelia* – PQUADEC, *Paramecium tredecaurelia* – PTRED, *Paramecium sonenborni* – PSON.

In order to find homologs of Spt4—the Spt5 partner—we searched the *P. tetraurelia* genome for genes encoding proteins similar to *S. cerevisiae* Spt4p using BLAST search (38). We identified four *SPT4* genes that share structural similarity with other eukaryotic Spt4 proteins, including Zn-finger motives and the Spt5 binding domain (Figure 1A). The four proteins can be divided into two families according to their evolutionary history and their expression profiles revealed by RNAseq analysis (42). Representatives of the first family are issued from the recent whole genome duplication (WGD) that shaped the *Paramecium* genome (43) and were named *SPT4mA* and *SPT4mB* as their expression is strongly induced during meiosis (‘m’ stands for ‘meiosis’). Genes belonging to the second family: *SPT4vA* and *SPT4vC*, are related to each other due to the intermediate WGD (‘A’ and ‘C’ in their names indicate that they are more distantly related than paralogs from the first family) and are vegetatively expressed (‘v’ for ‘vegetative’), however *SPT4vA* is expressed at a much higher level than *SPT4vC* (Figure 1B). Proteins encoded by the first family share high amino-acid identity—91%, while for the latter family there is only 74% identity. Both gene families are not related to each other by any known WGD event. Genes encoding proteins from both families—*SPT4m* and *SPT4v* – are present in all sequenced *Paramecium* species, each of them in one or two copies (Supplementary Figure S1) and (Figure 1C) (44,45). Analysis of their sequence similarity indicates that *SPT4* gene duplications in *Paramecium, Arabidopsis* and in *Oxytricha trifallax* occurred independently of each other. Moreover, the presence of meiosis-specific and vegetatively-expressed variants of Spt4 resembles the situation observed for Spt5 in *P. tetraurelia* – there we identified two distinct proteins, Spt5m and Spt5v, with different expression profiles, similar to those found now for Spt4m and Spt4v (21). At this point, we asked the question if Spt4mA/B and Spt4vA/C are counterparts of Spt5m and Spt5v, respectively?

### Overlapping localization patterns of Spt4vA and Spt4mB

**Figure 2.**
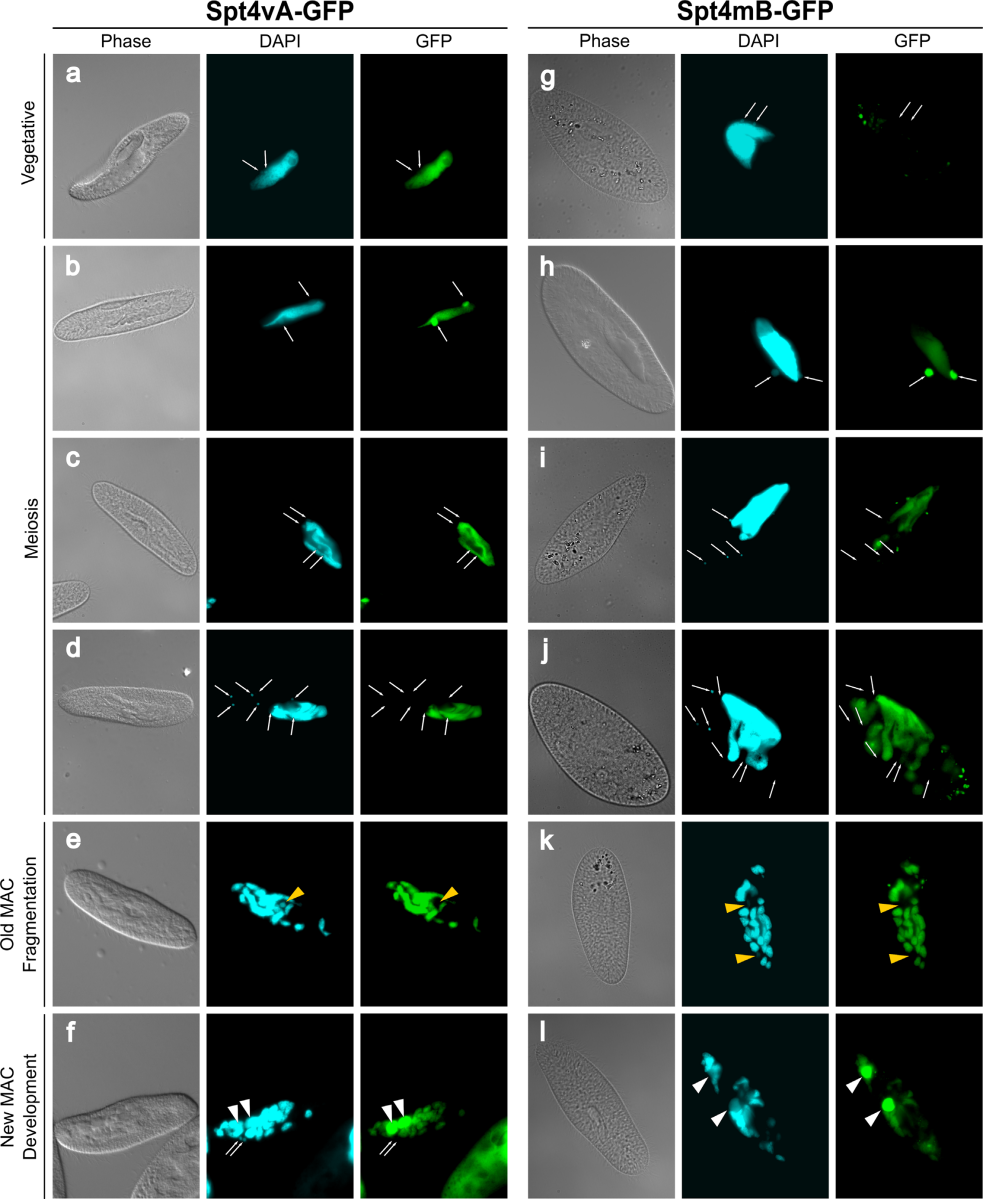
Localization of the SPT4vA-GFP and SPT4mB-GFP fusion proteins. Images were taken at significant stages of the life cycle: panels a and g – vegetative growth; panels b and h – beginning of meiosis; panels c and i – completion of I meiotic division; panels d and j – completion of II meiotic division; panels e and k – fragmentation of old MAC; panels f and l – development of new MACs. White arrows indicate MICs during vegetative growth and meiosis (on panels d and j not all MICs are visible but their localization was confirmed by photos taken at a different focus). Yellow arrowheads indicate the zygotic nucleus and division products of the zygotic nucleus while white arrowheads mark developing MACs.

In our previous study we showed that the two Spt5 homologs, Spt5m and Spt5v, are associated with distinct nuclei: the germline MIC during the sexual process and the somatic MAC, respectively. In order to check if Spt4 localization patterns match those described for Spt5, *Paramecium* cells were transformed with constructs encoding vegetative Spt4vA or meiosis-specific Spt4mB variants of Spt4 fused at the C-terminus with GFP. Localization of the fusion proteins expressed under control of the endogenous Spt4 promoters was registered by fluorescence microscopy at different stages of the life cycle—in vegetative cells and during the sexual process (Figure 2).

Consistently with the available RNAseq data (42), only the constitutively expressed variant Spt4vA-GFP was detected during vegetative growth in the maternal MAC, while Spt4mB-GFP was not observed (Figure 2, panels a and g). At the very beginning of the sexual cycle Spt4mB appears in meiotic MICs and in the maternal MAC. Spt4vA which is already located within the MAC also enters the MICs during meiosis (panels b and h). From this point both Spt4vA-GFP and Spt4mB-GFP share the same localization: they remain in the maternal MAC which is undergoing fragmentation and they disappear from the MICs before the end of the first meiotic division (panels c and i). Both proteins are not detected neither in the eight haploid products of the meiotic divisions (panels d and j), nor in the zygotic nucleus (panels e and k). As new MACs start to differentiate, the GFP signal for both proteins accumulates in new MACs (panels f and l). As we compare these results with the localization of the previously analyzed Spt5 homologs that were clearly specific either for somatic nuclei (Spt5v) or for germline nuclei (Spt5m) (21), we see a few important differences. First, despite the different localization patterns of Spt4vA and Spt4mB during vegetative growth, they are present in identical cellular compartments throughout the sexual cycle, suggesting that both Spt4 proteins may act in a redundant manner at this stage of the *Paramecium* life cycle. Moreover, both Spt4 proteins are absent from MICs undergoing meiotic divisions and from the zygotic nucleus and the products of its division, where we previously observed Spt5m. This raises the question whether there are different Spt5/Spt4 complexes present and active during vegetative growth and the sexual cycle.

### Silencing of all *SPT4* genes leads to high lethality of postsexual progeny

To investigate Spt4 function and to check possible redundancy between the *SPT4* genes, we silenced the *SPT4* genes either separately or together, two or three at a time, during vegetative growth and the sexual cycle. Since we used the ‘RNAi by feeding’ technique, silencing of two or three genes was achieved by mixing bacteria designed to silence individual genes. First, we studied the phenotypes of Spt4 depletion during vegetative growth. Neither silencing of *SPT4vC* nor double silencing of the *SPT4m* genes (*SPT4mA* and *SPT4mB*) influenced the rate of growth or cell shape/morphology. Each experiment in which *SPT4vA* was silenced – single silencing of *SPT4vA*, double silencing with *SPT4vC* (called later *Spt4v*-RNAi), or triple silencing of *SPT4vA* together with both *SPT4m* genes (called *SPT4*-RNAi) – resulted in a slightly lower number of divisions per day: ∼3.5 instead of 4 after >24 h of silencing (Supplementary Figure S2). However, cells silenced for any combination of *SPT4* genes were able to enter the sexual process of autogamy (self-fertilization) after a period of starvation. The obtained results indicate that Spt4 is not essential during vegetative growth and we were able to look for depletion phenotypes in sexual reproduction.

To check a possible role of Spt4 in sex-related processes, we silenced all *SPT4* genes, individually as well as in different combinations, and we observed the survival of sexual progeny following transfer of individual autogamous cells to standard medium (46). Silencing specificity and efficiency was confirmed by reverse transcription PCR (RT-PCR) (Supplementary Figure S3). Silencing of the genes *SPT4vA* and *SPT4vC* individually did not produce any visible phenotypes, while *SPT4mA* and *SPT4mB* gave partial phenotypes with significant lethality and survival rates of 57% and 33%, respectively (Table 1). Depletion of both Spt4m led to similar death rate as individual silencings and 46% surviving cells. The most prominent effect was obtained upon *SPT4*-RNAi (when all three significantly expressed *SPT4* genes were silenced)—most cell lines died and only 29% grew normally. We were surprised by the relatively high survival rate, so we decided to test the autogamy survivors for the MAC regeneration phenotype. Macronuclear regeneration is a self-recovery mechanism in *Paramecium*, in which cells incapable of producing functional new MACs retrieve maternal DNA and use it instead of new MACs (47). In order to study this phenotype, we developed a test based on cell lines transformed with *MSH2*-GFP constructs (46). *Paramecium* cells are not transformed permanently and typically loose the transgene after autogamy, together with fragments of the old somatic MAC, so that after the completion of a few vegetative divisions they do not express the GFP signal anymore. Moreover, Paramecium cells are incapable of entering autogamy again before completing 20 vegetative divisions (48). Vital postautogamous clones were analyzed under these two criteria. Cells that still expressed the GFP signal characteristic for the Msh2 transgene and were able to enter autogamy a few divisions after completing the previous one were counted as MAC regenerants. We found that cells classified as regenerants constituted the majority of the surviving progeny in *SPT4*-RNAi cells but were practically absent from *SPT4m*-RNAi cells as well as from the control RNAi (Table 1). We concluded therefore that the percentage of next generation progeny that successfully went through the sexual process in *SPT4*-RNAi experiment was overestimated by the pool of MAC regenerants. Taken together, simultaneous silencing of the three *SPT4* genes with high expression levels results in high lethality and only 8% of the progeny is able to produce a functional macronucleus. Our silencing experiments led to the conclusion that Spt4 is an important transcription elongation factor engaged in sexual reproduction. Moreover, it seems that *SPT4mA*, *SPT4mB* and *SPT4vA* are functionally redundant during the sexual process, while *Spt4vC*-RNAi gives no phenotype, neither in vegetative growth nor in the sexual cycle.

**Table 1.** Survival test of post-autogamous *SPT4*-silenced *P. tetraurelia* cells. The regeneration phenotype was tested in selected *SPT4* RNAi, *SPT4m* RNAi and *ICL* RNAi experiments marked by grey shading

Genes targeted by RNAi	*SPT4vA*	*SPT4vC*	*SPT4mA*	*SPT4mB*	*SPT4m*		*SPT4*		*ICL7*		*ND7*	None*
% wild type	90%	94%	57%	33%	46%	48%	29%	8%	97%	97%	90%	98%
% MAC regenerants	nt	nt	nt	nt	nt	0%	nt	19%	nt	1%	nt	nt
% sick	0%	1%	3%	4%	2%	10%	4%	6%	0%	0%	0%	0%
% death	10%	5%	40%	63%	52%	42%	67%	67%	3%	3%	10%	2%
Total cells	240	144	432	432	769	96	480	192	672	192	240	240
No. of experiments	5	3	9	9	12	2	8	4	11	4	5	5

*Standard *K. pneumoniae* medium.

*SPT4m = SPT4mA + SPT4mB*.

*SPT4 = SPT4mA + SPT4mB + Spt4vA*.

### Depletion of Spt4 affects the sRNA biosynthesis pathway

**Figure 3.**
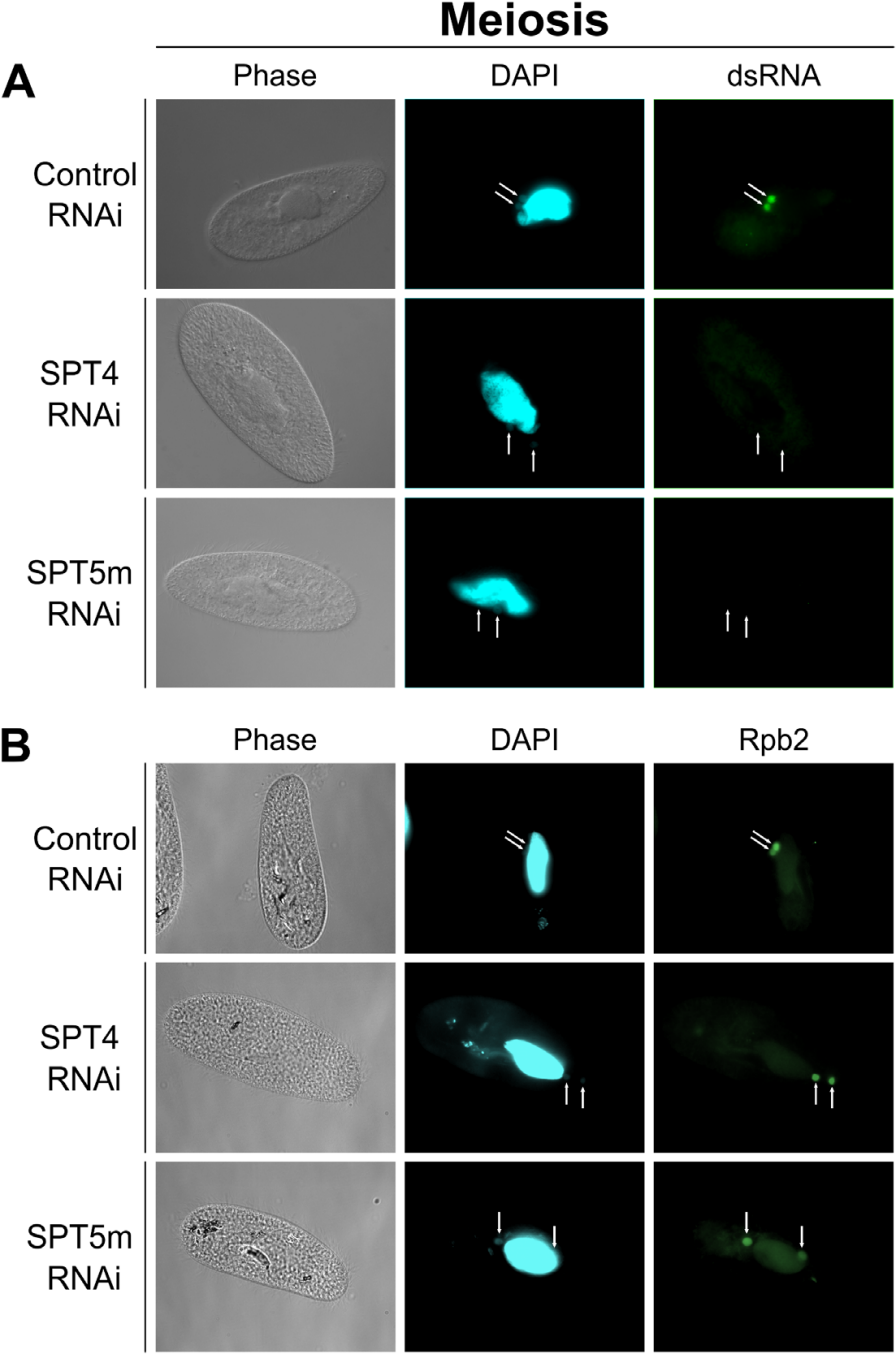
Transcriptional activity during meiosis. (**A**) Immunofluorescence detection of dsRNAs using the J2 antibody in cells silenced for *SPT4* and *SPT5m*. White arrows point to MICs at the beginning of the first meiotic division. (**B**) Immunofluorescence detection of RNA polymerase II subunit Rpb2-3xFLAG upon *SPT4* and *SPT5m* RNAi.

We showed recently that Spt5m influences the production of scnRNAs that have their origin in meiotic MICs (21). To verify if the elongation factor Spt4 shapes the landscape of these small regulatory RNAs, we performed a series of experiments. Since during early meiosis the genomes of germline nuclei are supposed to be bidirectionally transcribed giving rise to double-stranded RNA precursors of scnRNAs, we visualized dsRNA in the cell using the anti-dsRNA antibody J2 (Scicons). This monoclonal antibody specifically recognizes dsRNA of more than 40-bp length and it turned out to be useful for the detection of dsRNA by immunofluorescence throughout the sexual cycle of *Paramecium*. In the control cells, a specific anti-dsRNA signal was visible in the germline nuclei before the first meiotic division (Figure 3A). We did not detect any J2 signal in either the maternal MAC or the newly developing MACs, as was previously reported in *Tetrahymena* (49). In *Paramecium* there is no evidence for double-stranded RNA synthesis in an old MAC, while precursors of iesRNAs that are supposed to appear in new MACs are probably not abundant enough to be detected (26). In cells silenced for *SPT4* or *SPT5m* there was no signal corresponding to dsRNA in the meiotic MICs, indicating that both *SPT4-*RNAi and *SPT5m*-RNAi prevent dsRNA appearance, most probably by blocking all RNA synthesis in the germline nuclei. This is the first evidence that Spt4 influences the biogenesis of non-coding RNAs in a similar way as Spt5. Moreover, we checked localization of RNA polymerase II in meiotic cells using FLAG-tagged Rpb2 subunit. Rpb2 clearly co-localized with dsRNA in the germline nuclei before the first meiotic division and was also observed in somatic nucleus (Figure 3B). When we depleted Spt4 and Spt5m using RNAi, there we no visible change in Rpb2 localization, pointing rather to potential role of Spt4-Spt5 complex not in the RNA Pol II complex targeting to the meiotic MICs but polymerase activity at the germline genome.

**Figure 4.**
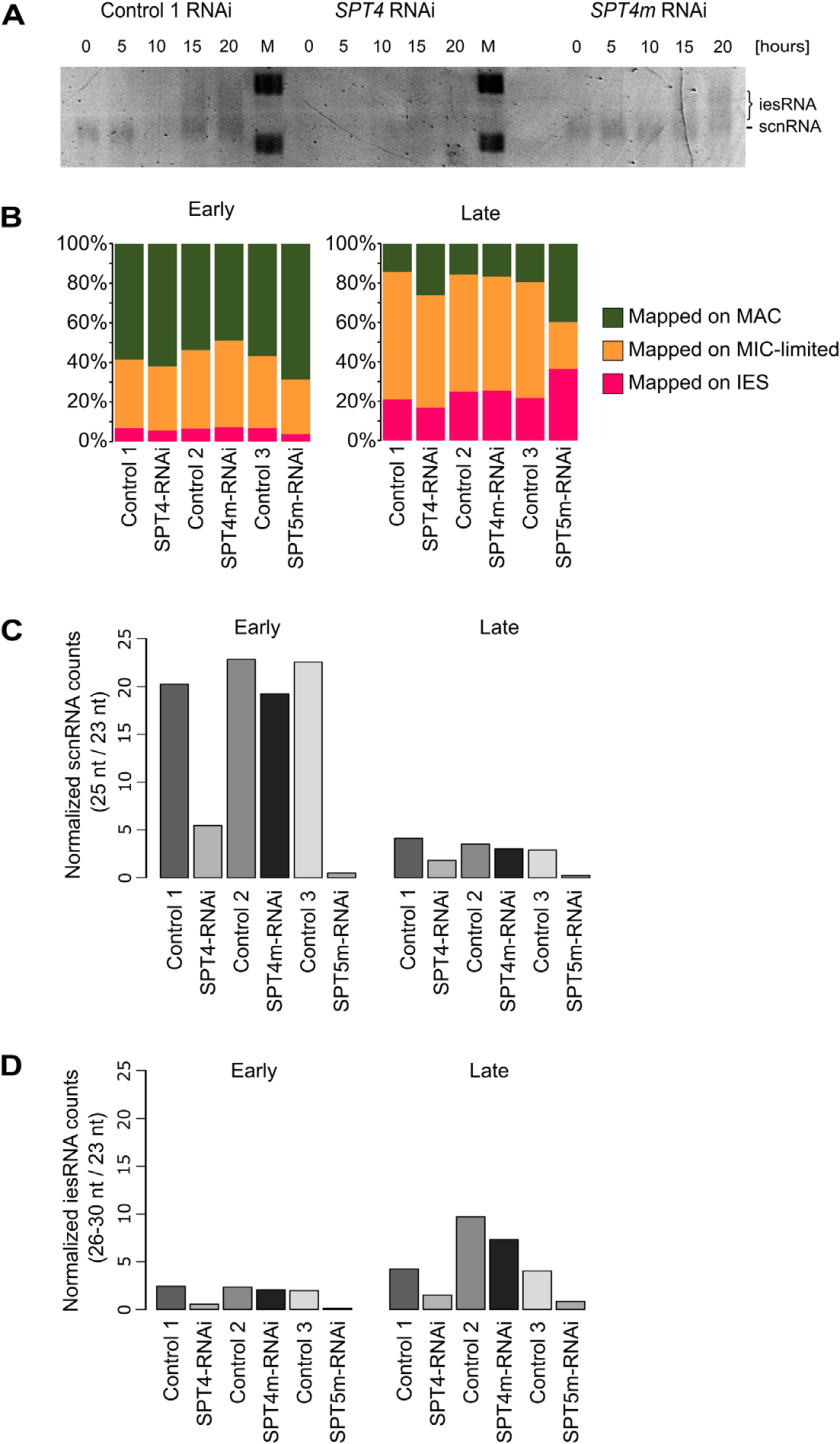
Analysis of small RNAs upon *SPT4* silencing. (**A**) SYBR Gold (Thermo Fisher Scientific) staining of sRNA separated by electrophoresis in a denaturing 15% polyacrylamide-urea gel. The T0 time point corresponds to an early stage of the sexual cycle (50% of cells with fragmented MAC, large fraction at meiosis), other time points are: 5, 10, 15, 20 h after T0, respectively. M: DNA Low Molecular Weight Marker (USB). (**B**) Histograms showing relative amounts of reads corresponding to MAC-specific, MIC-specific and IES sequences in particular samples. Control 1, 2 and 3 experiments were performed together with *SPT4*-RNAi, *SPT4m*-RNAi and *SPT5m*-RNAi, respectively. (**C** and **D**) Histograms showing sequence counts corresponding to scnRNAs and iesRNAs normalized to 23-nt siRNAs. Analyzed samples were: control 1 RNAi and *SPT4*-RNAi, control 2 RNAi and *SPT4m*-RNAi, as well as control 3 and *SPT5m*-RNAi from (21).

Next we performed *SPT4m-*RNAi, *SPT4-*RNAi and control silencing in a time-course experiment. Cell samples were collected at particular time points of the sexual cycle (Supplementary Figure S4). RNA isolated from all samples was separated by PAGE electrophoresis and stained by SYBR Gold. In control samples RNA bands corresponding to 25-nt scnRNAs were clearly visible for time points between T0 and T20 (Figure 4A). scnRNAs were also visible in samples isolated from cells subjected to *SPT4m*-RNAi, but they were not detectable in *SPT4*-RNAi. Smear signal corresponding to ∼26–30-nt iesRNAs were also noticeable in the T15 and T20 time points in control and *SPT4m*-RNAi, but not in *SPT4*-RNAi. In order to have a more accurate picture of the influence of *SPT4* genes on sRNA synthesis, we compared small RNA populations isolated at early (T0) and late (T15) stages of the sexual cycle from control, *SPT4m* and *SPT4* silencing experiments using high-throughput sequencing. RNA fractions ranging from 20- to 35-nt were excised from the gel and used for preparation of sequencing libraries by the TrueSeq Illumina kit. The obtained 20–30-nt Illumina sequencing reads were filtered for contaminations and rRNA and then mapped to the reference MAC genome, to MIC-specific sequences and to IES sequences (Supplementary Figure S5). The relative ratio of MAC-specific scnRNA to MIC/IES-specific scnRNA was calculated in all experimental conditions both at an early time point (T0) and at a late time point (T15). Counter selection of MAC scnRNAs occurred between the early and late time points in *SPT4m* and *SPT4* silencing, even though it seems to be delayed in the latter case, suggesting that triple silencing of *SPT4* genes impedes the scanning process to some extent (Figure 4B). Finally, the 25-nt read counts corresponding to scnRNAs and the 26–30-nt reads corresponding to iesRNAs were normalized using 23-nt endogenous siRNAs that are supposed not to be affected by the silencing. This procedure allowed us to compare the relative amounts between control and test conditions. Approximately 4-fold reduction of scnRNAs (at the early time point T0, when they are produced) and 3-fold diminution of iesRNAs (at the T15 time point) upon *SPT4*-RNAi were observed, whereas *SPT4m*-RNAi did not produce any significant changes in the levels of small non-coding RNAs (Figure 4C, D). Concluding, the silencing of three *SPT4* genes but not the silencing of two *SPT4m* autogamy-specific genes leads to a substantial reduction in scnRNA production, most probably due to a block in the synthesis of dsRNA scnRNA precursors.

### Spt4 contribution to the elimination of germline-specific DNA sequences

**Figure 5.**
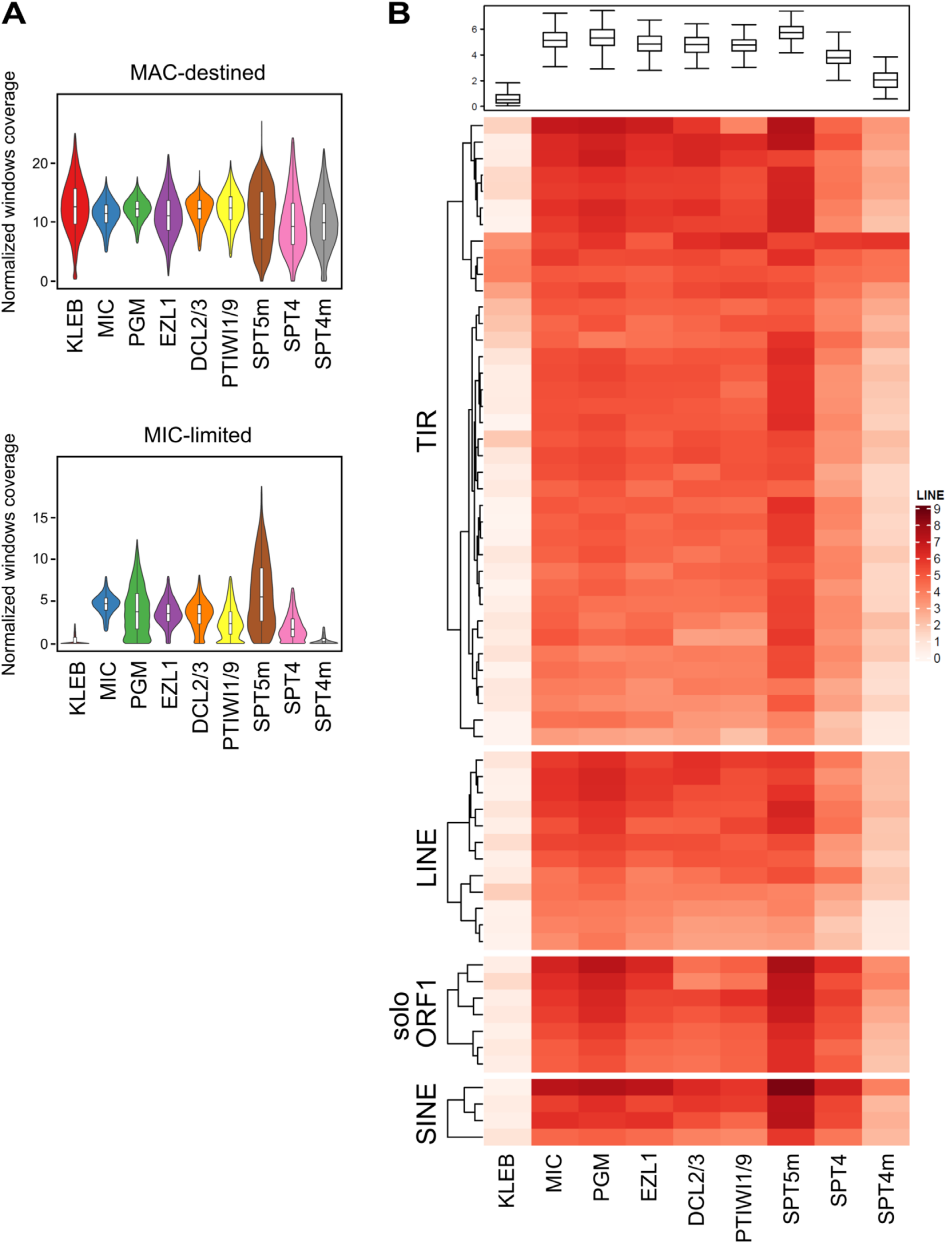
Retention of germline-specific DNA sequences upon *SPT4* silencing. (**A**) Violin plots showing coverage of MAC-destined and MIC-specific sequences upon silencing of *SPT4, SPT5m* and other genes from previous studies (25). KLEB is a reference experiment without silencing of any gene performed in standard *Klebsiella* medium, MIC corresponds to purified micronuclei. (**B**) Heatmap showing DNA coverage of *Paramecium* transposable elements after depletion of several genes. The intensity of the red coloring indicates the normalized coverage in RPKM (Reads per kilo base per million mapped reads) (log2). Each row represents a different TE consensus sequence, and the TE consensuses are ordered by hierarchical clustering within each TE family (LINE, TIR, Solo-ORF, SINE). The boxplots above the heatmap show the coverage (RPKM log2) for all TE.

The Spt5m protein is necessary for elimination of most germline-specific sequences during the development of a new MAC, so we wanted to check if Spt4 also plays a role in genome rearrangements. Since we saw no changes in DNA amplification levels in new MACs upon *SPT4*-RNAi and *SPT4m*-RNAi (Supplementary Figure S6), we decided to perform a genome-wide analysis of rearrangement patterns in new MACs. Genomic DNA was isolated from a fraction enriched for new, developing MACs, which was obtained from a culture at the end of the sexual cycle, and high-throughput sequencing and rearrangement analyses were performed, as described previously (24). Sequencing reads were afterwards mapped to a reference MAC genome, MIC-specific sequences and IES sequences. The obtained results showed that *SPT4m*-RNAi presents a phenotype close to the wild-type, while *SPT4*-RNAi leads to retention of MIC-specific sequences. However, we obtained much lower MIC coverage for *SPT4*-RNAi than for *SPT5m*-RNAi (Figure 5A). It must be also noted that the population of post-autogamous cells used for DNA extraction in the *SPT4*-RNAi experiment (but not in *SPT4m*-RNAi) might contain a significant fraction of MAC regenerants—the MAC regeneration level was estimated at 19% of the progeny (see Table 1). In MAC regenerants one large fragment of the old MAC grows and becomes the ‘new’ MAC which include a correctly rearranged version of the genome from the previous generation. This effect can significantly lower the fraction of reads mapping to MIC-specific sequences. However, a closer look at the retention of known transposable elements (TEs) revealed that Spt4 depletion leads to an elevated number of reads corresponding to DNA transposons (TIR) as well as LINE or SINE transposable elements (35). The effect observed for *SPT4*-RNAi was comparable with results obtained for the silencing of other components of the scnRNA pathway—Dcl2/3 (DICER like proteins), and we observed significant retention of all TEs (Figure 5B). Diminution of both Spt4m proteins resulted in much less reads mapping to TEs, leading to the conclusion that in this case the three *SPT4* genes act in a redundant manner.

To determine whether the retention of TEs can be associated with transcriptional upregulation of these elements, as previously reported upon *EZL1* silencing (37), we performed paired-end mRNA sequencing at late developmental stages (30–40 h after T0) in control, *SPT5m* and *SPT4*-RNAi conditions. Our analysis revealed that expression of TEs is up-regulated during MAC development upon *SPT5m*-RNAi and the effect was comparable with de-repression observed for *EZL1* (Supplementary Figure S7) (50). On the other hand, *SPT4*-RNAi resulted in a very small change in TEs expression. When *PGM* is silenced, we observe a retention of TEs but no de-repression of these elements, meaning that retention is not obligatory associated with non-deposition of histone marks (37). We postulate that Spt5m (and, to some extent Spt4) may be important for deposition of repressive chromatin marks that keep TEs silent in the early steps of the MAC development.

**Figure 6.**
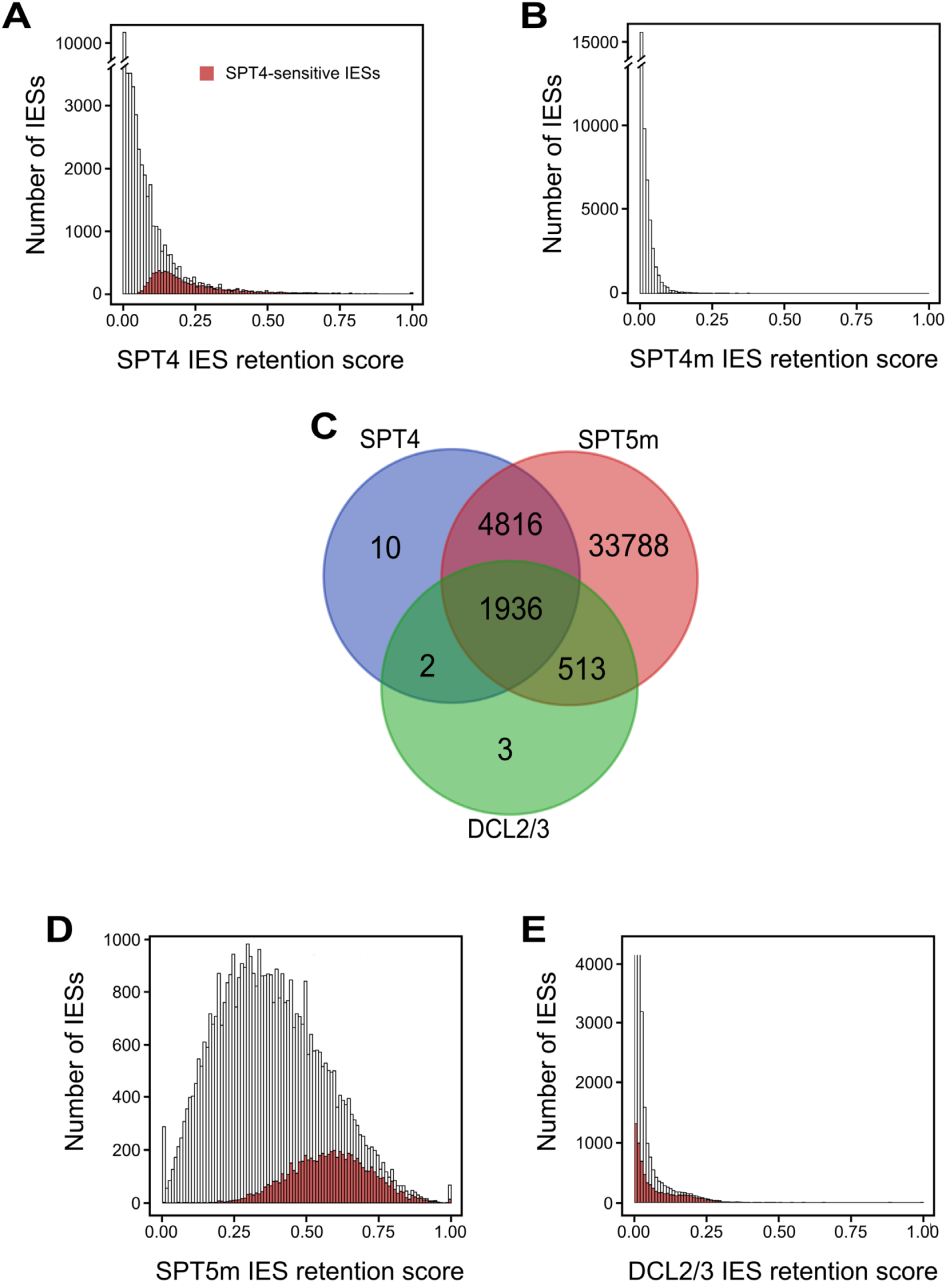
Analysis of IES retention in *SPT4* and *SPT4m* silencing experiments. (**A**) Histogram presenting distributions of IES retention scores for all IESs upon knockdown of *SPT4* genes. 6,764 IESs significantly retained in SPT4-RNAi are marked by coloring. (**B**)) Histogram displaying distributions of IES retention scores for all IESs in *SPT4m*-RNAi. (**C**) Venn diagram showing IESs affected by *SPT4*-RNAi, *SPT5m*-RNAi and *DCL2/3*-RNAi. (**D**, **E**) Histograms showing distributions of IES retention scores upon silencing of *SPT5m* or *DCL2/3*. IESs significantly affected by SPT4-RNAi are marked by coloring.

Analysis of IES excision revealed that *SPT4m*-RNAi practically does not influence excision of IESs—only ∼200 IESs were retained—while *SPT4*-RNAi causes significant retention of ∼6800 IESs—nearly 15% of all IESs. The retention scores (RS) for IESs that are *SPT4*-dependant are quite low (Figure 6A, B) and again this can be attributed to the presence of MAC-regenerants (described above) that can mask the influence of Spt4 on larger sets of IESs. Moreover, we did not observe any location preference for *SPT4*-dependent IESs—they are located both in coding and non-coding regions. Furthermore, there is no strong correlation between IES size and retention score—only a minor subset of ∼170 IESs larger than 1 kb show significantly higher mean retention scores than smaller IESs (Supplementary Figure S8). All IESs affected by Spt4 are included in a larger set of those retained by *SPT5m* RNAi (Figure 6C) and we observe that IESs sensitive to SPT4 show higher RS in *SPT5m* RNAi (Figure 6D). Similarly to the situation observed for *SPT5m*, over 70% of *SPT4*-dependent IES are not significantly retained in *DCL2/3*-RNAi. and seem to be unrelated to scnRNA biogenesis (Figure 6C, E). These results lead to the conclusion that Spt4 proteins, most probably along with Spt5m, could be involved in some unknown process that is necessary for the elimination of germline-specific sequences and is not dependent on scnRNAs produced by Dcl2/3 (21).

### Spt4/Spt5 complex formation

The localization of Spt4 homologs (Figure 2) and of Spt5 (21) during vegetative growth and the sexual cycle was substantial for the prediction of the composition of the Spt4/Spt5 complexes. In the MAC of the vegetative cell, formation of the complex is only possible for Spt5v and Spt4vA, while in the MAC during the sexual event pairing is probable between Spt5v and Spt4vA as well as between Spt5v and Spt4m. An Spt4/Spt5 complex in the meiotic MIC can be formed between Spt5m and both Spt4mB and Spt4vA, however only in a narrow time window, as co-localization occurs only right before the first meiotic division. On the other hand, all tested Spt5 and Spt4 proteins are present in the new MAC at the beginning of its development (Supplementary Figure S9).

As we were mostly interested in transcription of the germline genome in the MIC during meiosis, we decided to probe the composition of the Spt5/Spt4 complex characteristic to this nucleus.

In our first approach, in order to identify the interacting partner proteins, we used cell lines transformed with a transgene expressing either *SPT5m* or *SPT4mB* fused at its C-termini with the 3xFLAG tag. The control cells and transformed cells were grown and starved and then harvested at the beginning of the sexual cycle, when Spt5m and Spt4m are expressed. Proteins were extracted from the samples and affinity-purified using anti-FLAG antibodies. The isolated proteins were analyzed by Western blotting using anti-FLAG antibodies, confirming the presence of the fusion proteins in protein extracts from transformed cells (Supplementary Figure S10). Proteins co-purified with FLAG-tagged Spt5m and Spt4mB were studied by mass spectrometry in replicates (Supplementary Table S2). Experiments in which Spt4mB-3xFLAG was used as bait demonstrated its interaction with either Spt5m or Spt5v, which is consistent with the Spt4mB-GFP localization pattern (Table 2). Pairing of Spt4mB with Spt5m was detected less frequently than between Spt4mB and Spt5v (48 peptides versus 337), but this difference probably reflects the difference between the amounts of Spt4–Spt5 complex present in the MIC and in the MAC. Interestingly, mass spectrometry revealed that Spt5m-3xFLAG (which is present only in the MIC at the analyzed stage) interacts either with the meiotic Spt4mB or with Spt4vA. It confirmed one more time that both Spt4 variants act in a redundant manner. However, taking into account the number of experiments giving positive protein identification (7 out of 7 versus 4 out of 7) and the quantity of interacting peptides (14 versus 5 peptides), the data suggest that Spt5m may preferentially bind meiotic Spt4 (Table 2).

**Table 2.** Results of mass spectrometry experiments

Bait	Detected protein	Spt5m	Spt5v	Spt4mA/B	Spt4vA	Spt4vC	Ptiwi01/09
**Spt5m-3xFLAG**	No. of detected peptides	663	0	14	5	0	30
	Fraction of experiments	7/7	0/7	7/7	4/7	0/7	4/7
**Spt4mB-3xFLAG**	No. of detected peptides	48	337	391	0	0	8
	Fraction of experiments	5/5	5/5	5/5	0/5	0/5	4/5
**Control**	No. of detected peptides	0	0	0	0	0	5
	Fraction of experiments	0/7	0/7	0/7	0/7	0/7	1/7

**Figure 7.**
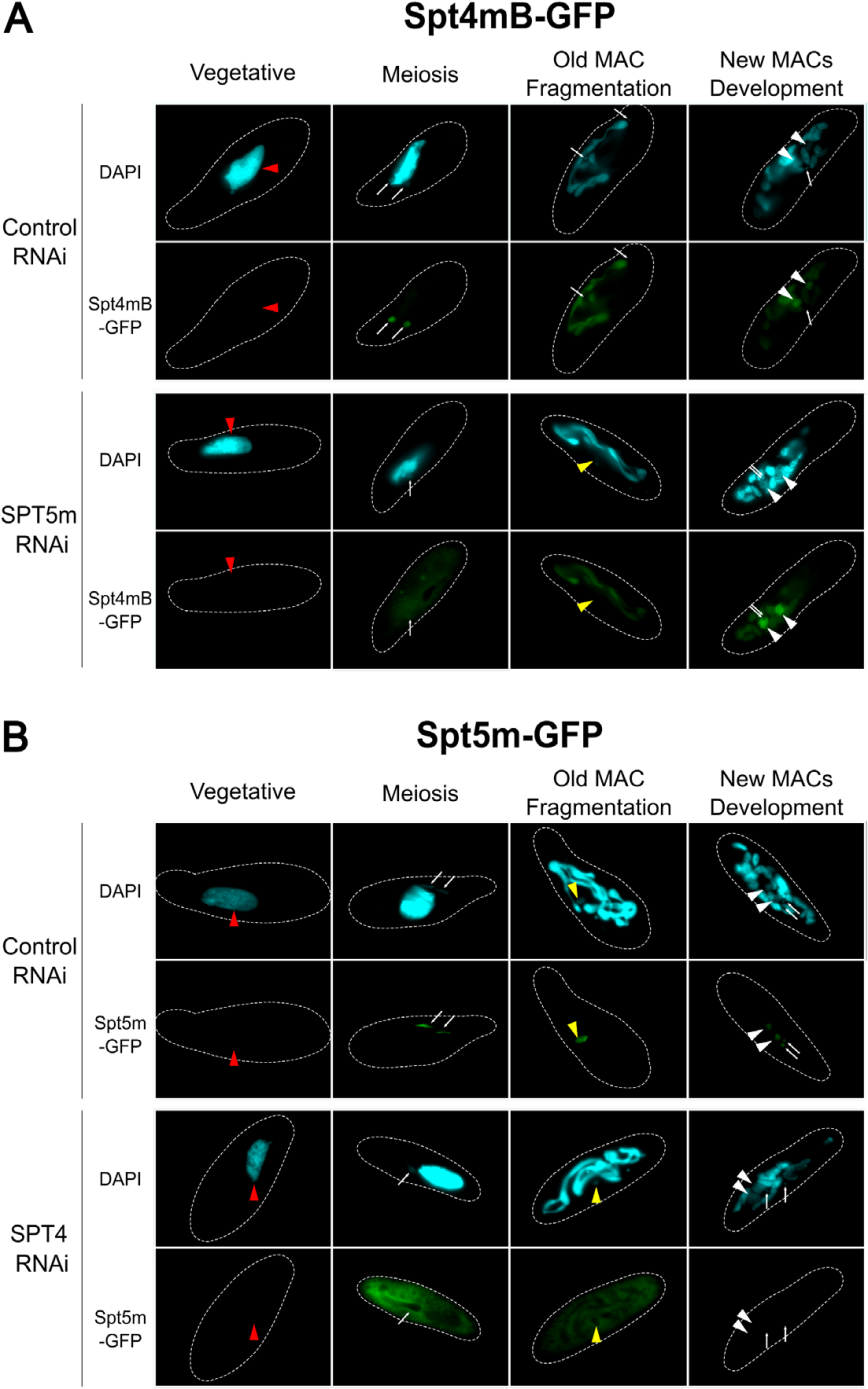
Localization of the Spt4mB and Spt5m proteins upon depletion of their respective putative partners. (**A**) Upon expression of a control RNAi, thesignal of Spt4mB-GFP is present in meiotic MICs, in the fragmented MAC and in the new MACs. Silencing of *SPT5m* distorts the localization of its partner exclusively during the initiation of the first meiotic division, causing dispersion of its signal across the cytoplasm. At later time-points normal localization is restored and the GFP signal can be found in the fragmented MAC and new MACs. (**B**) The localization pattern of Spt5m-GFP is unaltered upon expression of a control RNAi, whereas silencing of the *SPT4* genes prevents the entrance of Spt5m-GFP to the MICs and the new MAC throughout the whole sexual cycle of a cell. In both experiments images were taken during particular stages of the cell cycle: vegetative growth, initiation of the first meiotic division, fragmentation of the old MAC and development of the new MACs. Dashed white lines represent contours of the cells accordingly to the phase images. Red arrowheads point to maternal MACs, white arrows indicate MICs, yellow arrowheads mark zygotes and white arrowheads indicate new developing MACs.

Another attempt for the evaluation of the Spt4/Spt5 interaction was made by analyzing their localization upon depletion of the other partner. Cells expressing Spt4mB-GFP were silenced for *SPT5m* and observed throughout the sexual cycle using fluorescence microscopy. On the onset of meiosis the Spt4mB-GFP signal was dispersed in the cytoplasm and was not observed in the MICs. At later stages, the localization was normal – the GFP fusion protein was present both in fragments of the old MAC as well as in the new MACs (Figure 7A). These results show that reduced levels of Spt5m disturb the entrance of Spt4mB-GFP into MICs during the first meiotic division. In an analogical experiment we tested the localization of Spt5m-GFP upon *SPT4*-RNAi. The GFP signal was diffused across the cytoplasm throughout the sexual cycle and did not correlate with the nuclei – it was present neither in meiotic MICs, nor in developing MACs (Figure 7B). Taken together, this analysis pointed out that formation of the Spt4/Spt5 complex in the cytoplasm seems to be required for its entrance into germline nuclei during initial stages of the sexual cycle. Normal localization of Spt4mB-GFP in *SPT5m*-RNAi in later stages of the cycle may be explained by its interaction with Spt5v (which can be found in both maternal and developing MACs (21)) and by entrance of the Spt4mB–Spt5v complex into the nucleus. On the other hand, Spt5m showed non-nuclear localization throughout the entire sexual process upon *SPT4*-RNAi, even though a potential interaction between Spt5m and Spt4vA/B may perhaps drive the complex into the nucleus.

### PIWI entrance to the MAC is disrupted upon depletion of Spt4 and Spt5

**Figure 8.**
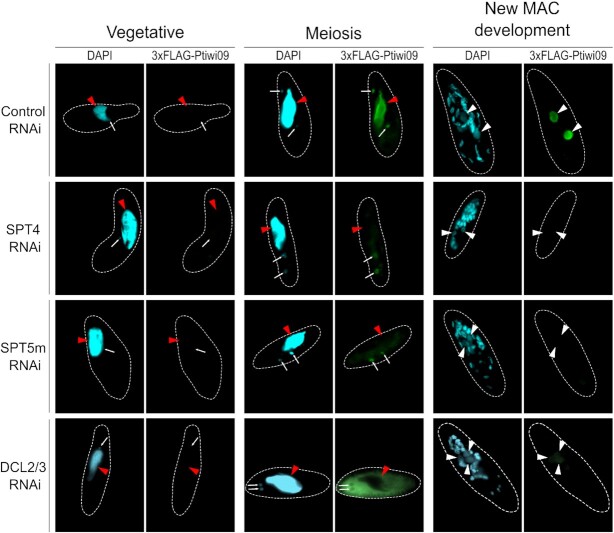
Localization of 3xFLAG-Ptiwi09 upon silencing of *SPT4*, *SPT5m* and *DCL2/3*. Ptiwi09 is expressed during meiosis and localizes to the MIC and the maternal MAC in the control experiment but is visible only in the MIC and cytoplasm after diminution of Spt4 or Spt5m. When *DCL2* and *DCL3* genes are silenced, 3xFLAG-Ptiwi09 is excluded from both MIC and maternal MAC. During the development of the new MACs 3xFLAG-Ptiwi09 is present in the new MACs in the control but is not detectable in *SPT4-RNAi* and in *SPT5m-RNAi* and hardly visible in DCL2/3-RNAi. The contours of cells are presented by dashed white lines, red arrowheads point to maternal MACs, white arrows designate MICs while white arrowheads mark new developing MACs. Not all MICs are visible. Three new MACs are present in the last cell in DCL2/3-RNAi panel.

Mass spectrometry analysis of proteins coimmunoprecipitated with Spt4 and Spt5 revealed the presence of peptides corresponding to the Piwi-like proteins Ptiwi01 and Ptiwi09 (Table 2). Assessment of these interactions was especially interesting because during *P. tetraurelia* development the Ptiwi proteins are involved in the pathway of small regulatory RNAs (51,52). Seeking further evidence for these interactions, we checked the localization of 3xFLAG-Ptiwi09 upon silencing of the expression of *SPT4* and *SPT5m*. Both conditions changed the localization of the Piwi-like protein in the same way. At the onset of the sexual process 3xFLAG-Ptiwi09 was present in the meiotic MIC in all experimental conditions, but later the protein was observed in control cells in the maternal MAC at the beginning of the sexual cycle and in the new MACs during the final stage of their development, while upon SPT4-RNAi and SPT5m-RNAi 3xFLAG-Ptiwi09 was absent from these locations (Figure 8). In order to avoid tag-specific bias the experiment was repeated using Ptiwi09 fused with a GFP tag and again the same influence of Spt4 and Spt5m depletion on Ptiwi09 localization was observed (Supplementary Figure S11). For this construct, however, localization in the meiotic MICs was not visible (as in a previous study (51)), probably due either to the lower sensitivity of a GFP fluorescence assay compared to an indirect immunofluorescence assay, or to the placement of the GFP tag after codon 116 of the PTIWI09 coding sequence compared to N-terminal position of the 3xFLAG. Still, the entire experiment demonstrated that absence of the Spt4/Spt5 proteins changes the fate of the Ptiwi09 protein – it can enter the MIC but is not transferred to the old and then to the new MAC. The character of this influence might be indirect and not requiring physical contact between Ptiwi01/09 and Spt4/5 proteins—the Spt4/Spt5 complex, as an upstream regulator of the scnRNA pathway, is necessary for the synthesis of scnRNAs and it is possible that only Ptiwi molecules that are bound to scnRNA are able to enter the macronucleus and act in the genome rearrangement pathway. This is supported by the fact that blocking scnRNA synthesis by depleting the DICER proteins Dcl2 and Dcl3 also prevented Ptiwi09 protein from entering the old and new MAC (Figure 8). We noticed however, that upon *DCL2/3* RNAi 3xFLAG-Ptiwi09 showed cytoplasmic localization at meiosis and was not observed in meiotic MICs. It cannot be explained easily, but there is a possibility that Ptiwi09 is transported to the germline nuclei along with Dcl2 and/or Dcl3.

## DISCUSSION

### Duplication of the genes encoding Spt4 and Spt5 in the course of neofunctionalization

Gene duplications and whole genome duplications are among the most important mechanisms driving the evolution of organisms. Most duplicated genes are lost, but some of them evolve by subfunctionalization or neofunctionalization toward new roles. Moreover, genes that encode proteins that form a complex usually evolve together and are kept in a stoichiometric number in the genome. The *Paramecium* genome was shaped by at least three whole genome duplications and contains an above average number of multigene families and therefore constitutes an excellent model for studies of gene evolution (43). Our research concerning the Spt4-Spt5 complex was indeed a study of two groups of paralogs—four paralogs of the *SPT4* gene and two of *SPT5*. The presence of more than one *SPT4* or *SPT5* gene is unusual in eukaryotes and can be observed in plants and some ciliates. *Paramecium* Spt5 proteins are specialized regarding their expression and localization patterns: Spt5v is expressed during vegetative growth and Spt5m was shown to be dedicated to meiosis and development (21). Spt4 proteins are encoded by two groups of paralogs resulting from WGD that show the same two different expression profiles—vegetative and peaking at meiosis but these groups are partially redundant regarding protein localization and function. It seems that Spt4vA, together with Spt5v, is responsible for expression of the somatic MAC genome, however Spt4 depletion doesn’t strongly affect vegetative growth, quite the opposite to Spt5v which is indispensable for cell growth. The non-essential role of Spt4 in the vegetative cycle is consistent with the results from *Saccharomyces cerevisiae* mutants (11). The situation gets complicated during the sexual process, when the germline genome is massively expressed during meiosis. According to our protein localization data, mass spectrometry analysis and silencing experiments not only Spt4mA/B but also Spt4vA, in cooperation with Spt5m, are responsible for RNA synthesis from the germline MIC and are essential during the sexual cycle. These observations are consistent with murine studies where a double knockdown of the *SPT4* ortholog SUPT4h led to the death of mice embryos (22). However, in a pulldown of proteins interacting with tagged Spt5m, even though peptides characteristic for both the vegetative and meiotic Spt4 protein were detected, we observed potential preferential binding of Spt4mA/B over Spt4vA. We conclude therefore that despite the redundancy among Spt4 homologs, Spt5m preferentially binds the meiotic version of Spt4. What we are facing here is therefore an overlapping of functions between Spt4m and Spt4v which had not gone through a complete neofunctionalization process like that observed for Spt5m and Spt5v. This resembles the situation in plants where one SPT5 protein (encoded by two genes) is part of the RNAPII elongation complex while the other SPT5-like protein KTF1/SPT5L has a role in RNAPV-dependent gene silencing, but two Spt4 proteins sharing ∼88% amino acid sequence identity (SPT4-1 and SPT4-2) are involved in both processes (53,54).

We noticed that *SPT4m* silencing leads to important lethality in postautogamous progeny, while the level of scnRNAs and iesRNAs and DNA elimination are hardly affected by *SPT4m*-RNAi. It is possible, that minor effect on genome rearrangements such as retention of a few IESs interrupting essential genes can lead to the cell death, but we cannot exclude the possibility that Spt4mA/B has a role other than DNA elimination during sexual reproduction. Spt4 factor that co-localize with a subset of kinetochore proteins, takes part in assembly of centromere heterochromatin and chromosome segregation in other organisms, may be important during meiosis in *Paramecium* and have an impact on the viability of a progeny (13).

### Spt4 as an important regulator of sRNA synthesis

**Figure 9.**
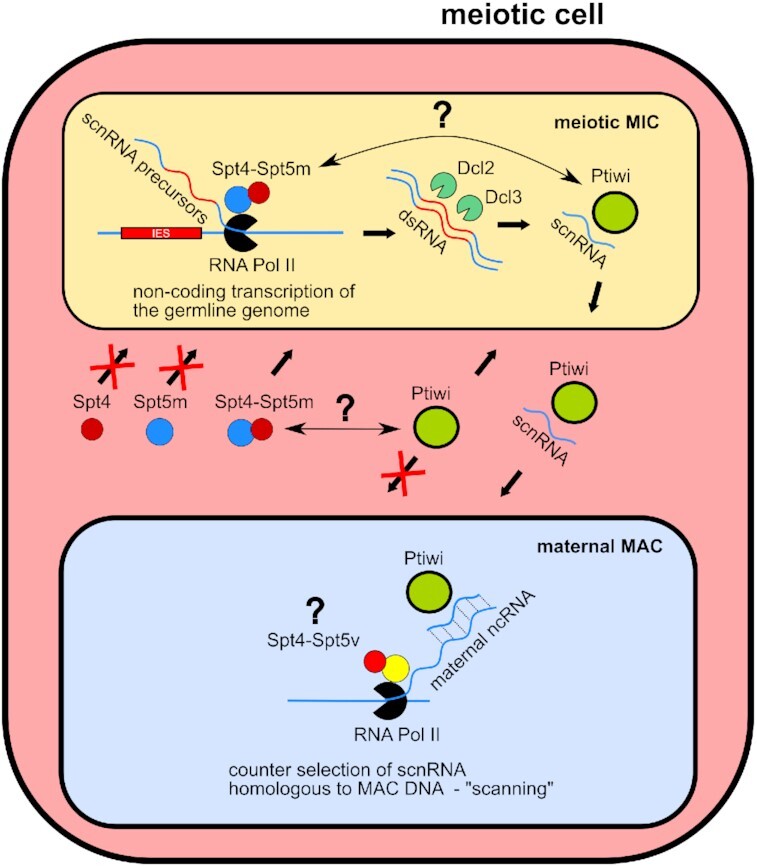
Role of the Spt4-Spt5m complex in the non-coding RNA pathway regulating DNA elimination. In cells depleted for Spt4 or Spt5m the remaining partner of the Spt4-Spt5m complex is not able to enter the germline nucleus. The Spt4-Spt5m complex most likely takes part in the synthesis of scnRNA precursors that can be detected as dsRNA and are processed into scnRNA. The Ptiwi01/09 proteins are loaded with scnRNA most probably in the MIC and are transferred to the maternal MAC where scnRNAs are compared with maternal transcripts representing the entire somatic genome. Silencing of *SPT4* or *SPT5m* causes dsRNA levels to drop below detection and leads to lower amounts of scnRNAs. It also blocks the transfer of the Ptiwi09 protein from the MIC into the maternal MAC, presumably due to the lack of scnRNA cargo.

According to the current model of RNA-dependent regulation of genome rearrangements in *Paramecium*, the initially inert germline nuclei undergo excessive transcription at the beginning of the sexual cycle, producing double-stranded RNA. These transcripts are thought to be subsequently processed by Dicer-like proteins into scnRNAs which coordinate the elimination of MIC-specific sequences from the developing somatic MAC (34). Our immunofluorescence analysis in which we used the J2 antibody to reveal dsRNA demonstrated, for the first time in *Paramecium*, that double stranded transcripts—possibly scnRNA precursors—are indeed present in MICs at an early stage of meiosis. These RNAs are affected by depletion of both Spt4 and Spt5m, suggesting a role of the MIC-specific Spt4-Spt5 complex in generalized expression of the germline genome. According to sRNA sequencing analysis, the impact of *SPT4*-RNAi on the biogenesis of mature scnRNAs is weaker than that of *SPT5m*-RNAi. This difference is surprising because silencing of *SPT4* blocks the entrance of Spt5m to the MIC and should therefore result in a full *SPT5m* phenotype (Figure 9). There are several possible explanations for these results. It is possible that the simultaneous silencing of three *SPT4* genes does not lead to a complete block of transcription, even though we observe significantly less mRNA for all silenced genes, and that the residual Spt4 molecules can partially fulfill their function. Also, one cannot exclude the hypothesis that even without the Spt4 cofactor some Spt5m molecules can enter the MIC and show enough activity to support transcription. It is also important to remember that the method we used for quantification of scnRNA levels is not very accurate and the exact impact of each silencing on scnRNA production is hard to estimate. Nonetheless, the observed effects demonstrate that both elongation factors, Spt4 and Spt5m, act as important regulators of small RNA synthesis during the sexual development of *P. tetraurelia*.

The presented results once again provide evidence for the hypothesis that the composition of the RNA Pol II complex in the germline nucleus is modified compared to the somatic complex. Such differences in the composition of the same complex, depending on the place of its action, may lead to different biological functions (55). According to the proposed model of genome rearrangements, all of the germline information needs to be transcribed during the initial stages of the sexual cycle (56). It is possible that such robust activity of RNA Polymerase II requires regulation by a distinct set of elongation factors than those operating within the somatic MAC. The discussed results are specific for *Paramecium*, but lines of evidence emphasizing the dissimilarity of somatic and germline transcription events exist likewise in other biological systems. For instance both in vitro and in vivo studies demonstrated that Primordial Germ Cells (PGCs) are found in a state of hypertranscription comparing to soma (57,58). Interestingly, upregulation of the PGCs transcriptome during embryogenesis in mice depends also on factors regulating pause release of RNA Polymerase II, indicating that our results reflect some globally conserved mechanisms (58).

### Spt4 and Spt5—do they act together or separately throughout the sexual process?

Spt4 and Spt5 form a complex that plays a central role in transcription and is responsible for promoter proximal pausing of RNA Pol II and for transcriptional elongation. The key function of the Spt4 subunit is to stabilize NGN domain of Spt5. As a result, most mutant Spt4 phenotypes may in fact be caused by Spt5 defects and can be attributed to the reduced structural stability of Spt5. It is however possible that both proteins can have independent roles.

In *P. tetraurelia*, we observed the Spt4 and Spt5m proteins simultaneously within the germline nuclei only right before and during the first meiotic division. This observation indicates that formation of their putative complex in the nucleus is possible exclusively during this stage. This phase is crucial for homologous recombination and, according to the literature, is associated with chromatin conformation changes as well as a shift in transcription output (59,60). Our experiments suggest that both the interaction between Spt5m and Spt4 and further dissociation of the complex may play a central role in the regulation of germline expression. Transition of the silent germline genome into a highly expressed genome resembles a phenomenon called hypertranscription which is observed during development in other organisms. Hypertranscription requires either increased RNA Pol II loading or a faster transcriptional rate (61) and both mechanisms can occur in the *P. tetraurelia* meiotic MIC. RNA Polymerase II is absent from the MIC during vegetative growth and needs to be recruited at meiosis while transcription of the entire genome may require a polymerase that is efficient and fast (34,62). It is probable that Spt5m forms a complex with Spt4, enters the germline nucleus and changes RNA Pol II properties toward generalized transcription of scnRNA precursors.

At later stages of the sexual process—during the second meiotic division and the formation and division of the zygotic nucleus—only Spt5m stays in the nuclei resulting from the germline nucleus. It is tempting to speculate that the disappearance of the Spt4 cofactor may have an important biological role in passing of the meiotic checkpoint or/and in switching off of transcription in the period between meiosis and formation of the new somatic nucleus. Dissociation of Spt4 from the complex or some process associated with it may block Spt5m, and perhaps RNA Pol II, on chromatin until the new MAC starts to differentiate and transcription needs to be reestablished.

Little is known about the regulation of transcription of the germline genome during meiosis in *Paramecium*. Protein phosphorylation is a process that was shown to control transcription on multiple levels and it is possible that it is responsible for the regulation of germline transcription in *Paramecium*. Phosphorylation of Spt5 recruits chromatin remodelers, introducing the H3K4 methylation mark, coupling transcription elongation with chromatin modification that is associated with resolving the topological structures of chromatin (63,64). However, other studies showing a role of Spt5 in early transcription proved that neither the Spt5 CTR (C’-terminal repeats) domain, which can be phosphorylated, nor the Spt4 partner are important for promoter escape (65). Interestingly, Spt4 was shown to play a role in autophagy as an inhibitor of Spt5 phosphorylation (16) and it is possible that it is also necessary for the progression of meiosis. This putative role of Spt4 is especially interesting because of the evidence indicating that dephosphorylation of Spt5 decreases the elongation rate of the RNA Pol II complex (66). It is possible that Spt4 may function in the inhibition of Spt5m phosphorylation and in consequence may influence the extensive recruitment of the Pol II complex to the germline genome. Subsequent phosphorylation may result in a synchronous increase in the elongation rate of all recruited RNA Pol II complexes, leading to hypertranscription of germline nuclei at the initial stage of meiosis. Nevertheless, both Spt5 variants in *Paramecium* lack the CTR domain which is phosphorylated in other organisms and their phosphorylation state has not been studied yet (21).

### Relation between the Spt4-Spt5m complex and other components of the sRNA pathway—Dcl2/3 and PIWI

The putative role of the Spt4–Spt5m complex in generalized transcription of the germline places it at the first step of the scnRNA-dependent pathway regulating genome rearrangements in *Paramecium*. According to the current model, double-stranded scnRNA precursors are processed by Dcl2 and Dcl3 (26,34) and loaded onto Ptiwi01/09 (51,52). Transcription, processing and, taking into account our observations of Ptiwi09 localization, most probably Ptiwi loading with scnRNAs, take place in the meiotic MIC (Figure 9). One would expect that any disruption of the pathway at this early stage should result in the same phenotype regarding genome rearrangements of the new MAC, but this is not the case: we clearly see that silencing of *SPT5m* and *SPT4* affects IES sequences that are not dependent on Dicer proteins. This is apparent even though in the case of *SPT4* the proportion of affected germline sequences was much smaller than for *SPT5m*: *SPT4*-RNAi significantly affects only 15% of IESs while *SPT5m*-RNAi impacts practically all IES sequences. We think that the *SPT4* phenotype we observed was weaker due to the fact that our samples contained DNA also from MAC regenerants. One possible explanation for the fact that Spt4/Spt5m affects a larger group of IESs would be that Spt4/Spt5m-dependent transcripts are processed by a Dicer-independent pathway to some unknown sRNAs that are important for the excision of selected IESs. The biogenesis model of other meiosis-specific small RNAs has been lately revisited, indicating that regulatory transcripts may emerge through distinct pathways within a single organism (67). It is also probable that the Spt4–Spt5m complex plays a role that is necessary for genome rearrangements and is distinct from the scnRNA pathway. For the moment however we are not able to identify this function.

Unexpectedly, Ptiwi01/09 was identified as a putative partner of both Spt4mA and Spt5m in mass spectrometry analyses of pulldown experiments. However, the specificity of these interactions was challenged by the identification of Ptiwi peptides within one control sample and by the fact that Ptiwi proteins seem to be very abundant in the cell. Nevertheless, our immunofluorescence assays showed that activity of Spt5m and Spt4 is necessary for the transfer of Ptiwi09 from the meiotic MIC to an old MAC and, subsequently, to a new MAC. Taking into account that both proteins forming the Spt4/5 complex are necessary for the biosynthesis of RNA precursors within meiotic MICs and that their depletion reduces formation of mature scnRNAs, it is possible that Ptiwi09 entering germline upon *SPT4*/*SPT5m* knockdown cannot be loaded with sRNAs and subsequently transferred into the maternal MAC, as PIWI/Argonaute localization depends on its cargo. Indeed, when scnRNA synthesis was blocked by the absence of the DICER proteins Dcl2 and Dcl3, Ptiwi09 was not able to enter the old and new MAC. Moreover, the fact that in an sRNA sequencing experiment we observe a delay in the selection of MIC-specific scnRNAs may be explained by the influence of SPT4/SPT5m-RNAi on Ptiwi transfer to an old MAC, where the scanning process takes place. These experiments provide evidence for a mutual relation between Ptiwi proteins and Spt4/Spt5m, however they give no proof for a physical interaction (Figure 9). It was shown in *Arabidopsis* that KTF1/SPT5L interacts with the AGO4 protein, but in this case the KTF1 protein contains C-terminal WG/GW motifs known to be responsible for Argonaute binding in eukaryotes (68). The proposed model at least partially explains the observed relation, placing Spt4 and Spt5m as upstream regulators of Ptiwi, which correlates with the model proposed in *C. elegans* which suggests a direct role of a factor regulating Pol II processivity in the biogenesis of small RNAs associated with PIWI proteins (69).

The results presented in this study prove not only that Spt4 affects the levels of small RNAs produced during the sexual cycle but also that together with Spt5 it regulates the functioning of downstream factors engaged in their pathway. The nature of the above mentioned relationship needs further elucidation but it is tempting to think that its clarification will bring new answers about the biogenesis of small regulatory transcripts (70). Further research on the Spt4/5 complex may shed new light on meiotic transitions and help us understand the role of epigenetic mechanisms during reproduction.

## DATA AVAILABILITY

ParameciumDB accession numbers of studies genes are as follows: PTET.51.1.G0010243 – *SPT4mA*, PTET.51.1.G0080193 – *SPT4mB*, PTET.51.1.G0320167 - *SPT4vA*, PTET.51.1.G0130177 – *SPT4vC*, PTET.51.1.G0480005 – *RPB2*.

Sequencing data underlying this article are available in the European Nucleotide Archive using the accession number PRJEB44415.

The mass spectrometry proteomics data have been deposited to the ProteomeXchange Consortium via the PRIDE (71) partner repository with the dataset identifier PXD027748 and 10.6019/PXD027748.

## Supplementary Material

gkac106_Supplemental_File
