# Supplementary material for: The transient Spt4-Spt5 complex as an upstream regulator of non-coding RNAs during development
Source: Nucleic Acids Res. 2022 Feb 21;50(5):2603–20. doi: 10.1093/nar/gkac106 (PMC8934623; doi:10.1093/nar/gkac106)
Supplement: gkac106_Supplemental_File [file gkac106_supplemental_file.pdf]

Supplementary information for

The transient Spt4-Spt5 complex as an upstream regulator of non-coding RNAs during development

Dawid Owsian, Julita Gruchota, Olivier Arnaiz, and Jacek K. Nowak

Supplementary Figures

Supplementary Tables

Supplementary Figures

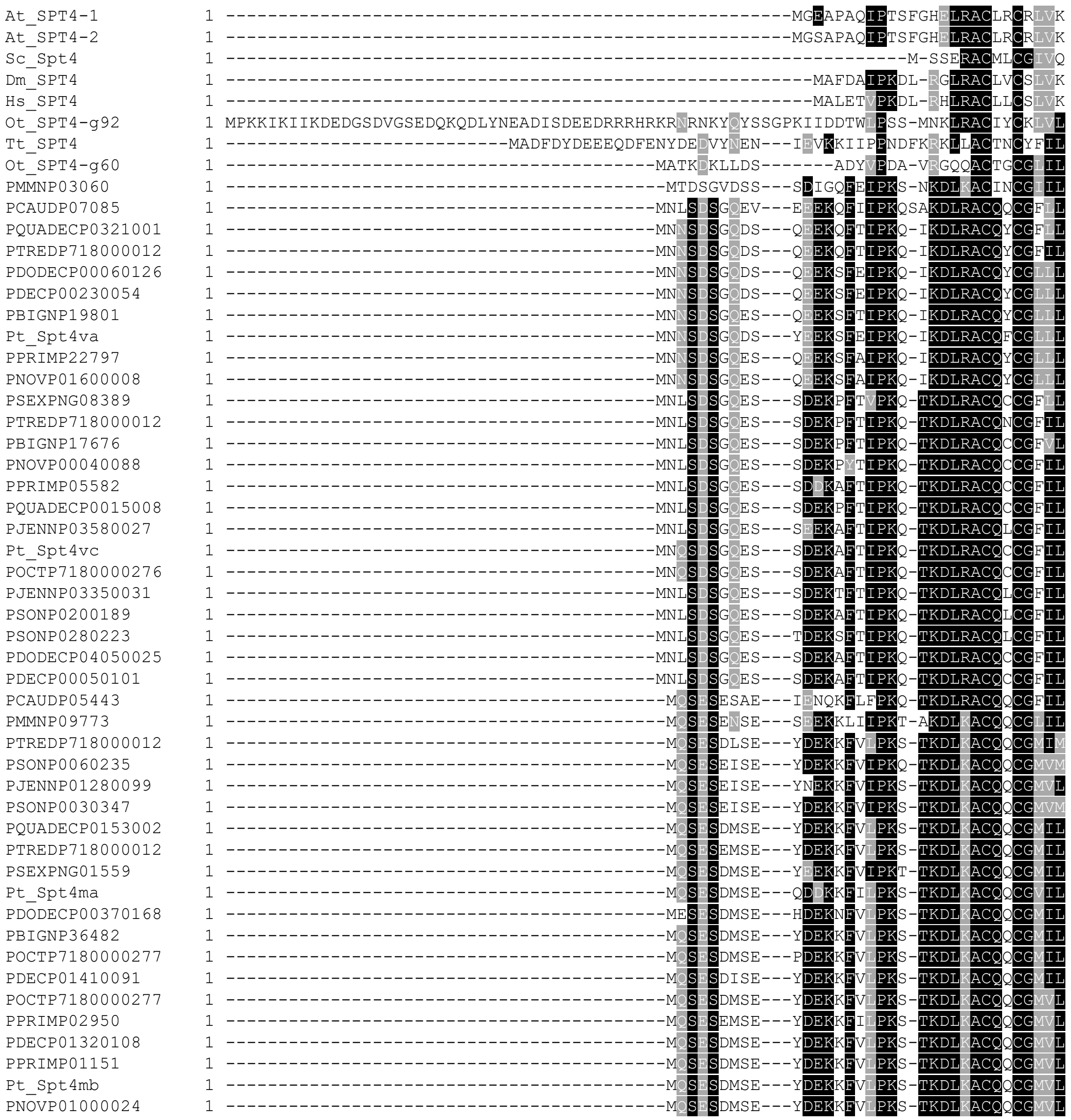

```
At_SPT4-1          27 TYDQFRD--SGCENCP-FFKIEDDHERIVDVTTPNFNGIISMMDPRRSWAAR-WLRI---GKFAPGCYTLAVSEALPE--
At_SPT4-2          27 TYDQFRD--AGCENCP-FFKMEEDHERIVEVTTPNFNGIISVMDPSRSWAAR-WLRI---GKFAPGCYTLAVSEPLPE--
Sc_Spt4            15 TTNEFNR--DGCPNCQGIFEEAGV--STMECTSPSFEGLVGMCKPTKSWVAK-WLSV---DHSIAGMYAIKVDGRLPA--
Dm_SPT4            24 SFDQFET--DGCENCEEFLRMKNNKDNVYDHTSNNFDGIIALTTPTDSWVAK-WQRL---SRFTRGIYAISVSGTLPQ--
Hs_SPT4            24 TIDQFEY--DGCDNCDAYLQMKGNREMVYDCTSSSFDGIIAMMSPEDSWVSK-WQRV---SNFKPGVYAVSVTGRLPQ--
Ot_SPT4-g92        80 NQEKWSK-LKQCPNCPES-------SASLDETTDNYESLISLVLPLKSWVAQ-WQQM---RNCIPGIYAMAVRAPN----
Tt_SPT4            51 TQEQWRD-YQNCPNCDVS-------SN-NPKPTAKFTGITCITDPAKSWQTNRMRIVKRKDVKVPGAYAVNINEDYDYQQ
Ot_SPT4-g60        32 TSSQWKE-NGSCPNCKDE-------A--HLETTNDFGGVVALMHPKESWVAK-WNNF---TKFVPGLYAIQFQEHNQYNP
PMMNP03060         35 NSDQWKNQKFKCVNDCGE-----------NEPTRSFSGIVCLMKPSKSWVAKKLGHA---RDIKPGLYAIDVQAE-----
PCAUDP07085        37 TQDQWNK-NNQCPNDCNA-----------A-QTKLYSGVVCLMKPSKSWVVRKLGIP---RNIHPGLYAIDVQAD-----
PQUADECP0321001    36 TLEQWNK-INQCLNGCSA-----------D-QTKIFSGVICVMKPSKSWVIKKLGNS---KNIQPGLYAIDVQAE-----
PTREDP718000012    36 TLEQWNK-INQCLNGCSA-----------D-QTKLFSGVICVMKPSKSWVIKKLGNS---KNIHPGLYAIDVQAE-----
PDODECP00060126    36 TLEQWNK-TSQCLNGCSA-----------D-QTKIYSGVICVMKPSKSWVIKKLGNS---QNIHPGLYAIDVQAE-----
PDECP00230054      36 TLEQWNK-ISQCLNGCSA-----------D-QTKIYSGVICVMKPSKSWVIKRLGIS---QNIHPGLYAIDVQAE-----
PBIGNP19801        36 TLEQWNK-ITQCLNGCSA-----------D-QTKLYSGVICVMKPSKSWVIKKLGIS---KNIHSGLYAIDVQAE-----
Pt_Spt4va          36 TLEQWNK-ITQCLNGCSA-----------D-QTKIYSGVICVMKPSKSWVIKKLGNS---KNIHPGLYAIDVQAE-----
PPRIMP22797        36 TLEQWNK-ITQCLNGCSA-----------D-QTKLFSGIICVMKPSKSWVIKKLGNS---KNIHPGLYAIDVQAE-----
PNOVP01600008      36 TLEQWNK-ITQCLNGCSA-----------D-QTKIYSGVICVMKPSKSWVIKKLGNS---KNIHPGLYAIDVQAE-----
PSEXPNG08389       36 TQEQWNK-NNQCLNGCSA-----------D-QTKLFSGVICIMKPSKSWAIKRLGIP---RNIHPGLYAIDVQAD-----
PTREDP718000012    36 TSEQWNK-NSQCYNGCSA-----------D-QTKLFTGVICVMKPSKSWVIKKLGNQ---KNIHPGLYAIDLQAD-----
PBIGNP17676        36 TQEQWNK-NSQCLNGCSA-----------D-QTKLFTGVICVMKPSKSWVVKKLGNQ---KSIHPGLYAIDVQAD-----
PNOVP00040088      36 TQEQWNK-NSQCLNGCSA-----------D-QTKLFTGVICVMKPSKSWVVKKLGNQ---KNIHPGLYAIDVQAD-----
PPRIMP05582        36 TQEQWNK-NSQCLNGCSA-----------D-QTKLFTGVICVMKPSKSWVVKKLGNQ---KNIHPGLYAIDLQAD-----
PQUADECP0015008    36 TQEQWNK-NSQCLNGCSA-----------D-QTKLFTGVICVMKPSKSWVVKKLGNQ---KNIHPGLYAIDLQAD-----
PJENNP03580027     36 TQEQWNK-NSQCLNGCSA-----------D-QTKLFSGVICVMKPTKSWVVKKLGNQ---RNIHPGLYAIEVQAD-----
Pt_Spt4vc          36 TQEQWNK-NSQCFNGCSA-----------D-QTKLFTGLICVMKPSKSWVVKKLVNQ---QNIHPGLYAIDLQAD-----
POCTP7180000276    36 TQEQWNK-NSQCLNGCSA-----------D-QTKLFTGIICVMKPSKSWVVKKLGNQ---QNIHPGLYAIDLQAD-----
PJENNP03350031     36 TQEQWNK-NSQCLNGCSA-----------D-YTKIFTGVICVMKPSKSWVVKKLGNQ---RNIHPGLYAIDVQAD-----
PSONP0200189       36 TQEQWNK-NSQCLNGCSA-----------D-YTKIFTGVICVMKPSKSWVVKKLGNQ---RNIHPGLYAIDVQAD-----
PSONP0280223       36 TQEQWNK-NSQCLNGCSA-----------D-YTKIFTGVICVMKPSKSWVVKKLGNQ---RNIHPGLYAIDVQAD-----
PDODECP04050025    36 TQEQWNK-NSQCLNGCSA-----------D-QTKIFTGVICVMKPSKSWVVKKLGNQ---QNIHPGLYAIDVQAD-----
PDECP00050101      36 TQEQWNK-NSQCLNGCSA-----------D-QTKIFTGVICVMKPSKSWVVKRLGNQ---QNIHPGLYAIDLQAN-----
PCAUDP05443        35 TQEQWNK-LSECPNDCNA-----------S-QTKLFSGLICVMKPQKSWVMRKLGNP---KNVCPGLYALDVQAE-----
PMMNP09773         35 TSEQWNR-ERECFNGCNS-----------G-QTKSFSGMICVLKPQKSWVMRKLGNP---SNVYPGLYAIDVQAD-----
PTREDP718000012    35 TIEQWNR-EIECPNQCNA-----------T-QTKLFSGIICVLKPSQSWVMRKLGNP---RNIHPGLYAIDVQAD-----
PSONP0060235       35 TMEQWNR-EVECPNSCNA-----------S-QTKLFSGLICVLKPSQSWVMRKLGNP---RSIHPGLYAIDVQAD-----
PJENNP01280099     35 TIEQWNR-EVECPNSCNA-----------S-QTKLFSGLICVLKPSQSWVMRKLGNP---RSIHPGLYAIDVQAD-----
PSONP0030347       35 TIEQWNR-EVECPNSCNA-----------S-QTKLFSGLICVLKPSQSWVMRKLGNP---KSIHPGLYAIDVQAD-----
PQUADECP0153002    35 TVEQWNR-QVECPNACNS-----------S-QTKLFSGIICVLKPSQSWVMRKLGNP---RNIHPGLYAIDVQAD-----
PTREDP718000012    35 TVEQWNR-QVECPNTCNS-----------S-QTKLFSGMICVLKPSQSWVMRKLGNP---RNIHPGLYAIDVQAD-----
PSEXPNG01559       35 TIEQWNR-EVECPNSCNA-----------S-QTKLFSGIICVLKPSQSWVMRKLGNP---RSIHPGLYAIDVQAD-----
Pt_Spt4ma          35 TVEQWNR-AVECPNSCNA-----------S-QTKLFSGMICVLKPSQSWVMRKLGNP---RSIHPGLYAIDVQAD-----
PDODECP00370168    35 TVEQWNR-EAECPNSCNA-----------S-QTKLFSGMICVLKPSQSWVMRKLGNP---RSIHPGLYAIDVQAD-----
PBIGNP36482        35 TVEQWNR-EVECPNSCNT-----------S-QTKLFSGMICVLKPSQSWVMRKLGNP---RSIHPGLYAIDVQAD-----
POCTP7180000277    35 TVEQWNR-EVECPNSCNA-----------S-QTKLFSGMICVLKPSQSWVMRKLGNP---RSIHPGLYAIDVQAD-----
PDECP01410091      35 TVEQWNR-EVECPNSCNA-----------S-QTKLFSGMICVLKPSQSWVMRKLGNP---RSIHPGLYAIDVQAD-----
POCTP7180000277    35 TVEQWNR-EAECPNSCNA-----------P-QTKLFSGMICVLKPSQSWVMRKLGNP---KSIHPGLYAIDVQAD-----
PPRIMP02950        35 TVEQWNR-EVECPNSCNA-----------S-QTKLFSGMICVLKPSQSWVMRKLGNP---KNIHPGLYAIDVQAD-----
PDECP01320108      35 TVEQWNR-EVECPNSCNA-----------S-QTKLFSGMICVLKPSQSWVMRKLGNP---RSIHPGLYAIDVQAD-----
PPRIMP01151        35 TVEQWNR-EVECPNSCNA-----------S-QTKLFSGMICVLKPSQSWVMRKLGNP---KNIHPGLYAIDVQAD-----
Pt_Spt4mb          35 TVEQWNR-EVECPNSCNA-----------S-QTKLFSGMICVLKPSQSWVIRKLGNP---KSIHPGLYAIDVQAD-----
PNOVP01000024      35 TVEQWNR-EVECPNSCNA-----------S-QTKLFSGMICVLKPSQSWVMRKLGNP---KSIHPGLYAIDVQAD-----
```

```
At_SPT4-1         98 EMQFICQQARVQYVPPKRI---
At_SPT4-2         98 EMQHLCQEERVQYVLPKRM---
Sc_Spt4           85 EVVELLP----HYKPRDGSQVE
Dm_SPT4           96 STLRDMKNRGIVYKSRDRSQR-
Hs_SPT4           96 GIVRELKSRGVAYKSRDTAIKT
Ot_SPT4-g92          ----------------------
Tt_SPT4          122 DTFE------------------
Ot_SPT4-g60       98 ENEYEMNDDE------------
PMMNP03060           ----------------------
PCAUDP07085          ----------------------
PQUADECP0321001      ----------------------
PTREDP718000012      ----------------------
PDODECP00060126      ----------------------
PDECP00230054        ----------------------
PBIGNP19801          ----------------------
Pt_Spt4va            ----------------------
PPRIMP22797          ----------------------
PNOVP01600008        ----------------------
PSEXPNG08389         ----------------------
PTREDP718000012      ----------------------
PBIGNP17676          ----------------------
PNOVP00040088        ----------------------
PPRIMP05582          ----------------------
PQUADECP0015008      ----------------------
PJENNP03580027       ----------------------
Pt_Spt4vc            ----------------------
POCTP7180000276      ----------------------
PJENNP03350031       ----------------------
PSONP0200189         ----------------------
PSONP0280223         ----------------------
PDODECP04050025      ----------------------
PDECP00050101        ----------------------
PCAUDP05443          ----------------------
PMMNP09773           ----------------------
PTREDP718000012      ----------------------
PSONP0060235         ----------------------
PJENNP01280099       ----------------------
PSONP0030347         ----------------------
PQUADECP0153002      ----------------------
PTREDP718000012      ----------------------
PSEXPNG01559         ----------------------
Pt_Spt4ma            ----------------------
PDODECP00370168      ----------------------
PBIGNP36482          ----------------------
POCTP7180000277      ----------------------
PDECP01410091        ----------------------
POCTP7180000277      ----------------------
PPRIMP02950          ----------------------
PDECP01320108        ----------------------
PPRIMP01151          ----------------------
Pt_Spt4mb            ----------------------
PNOVP01000024        ----------------------
```

Figure S1. Multiple alignment of Spt4 proteins present in model organisms and in sequenced *Paramecium* species performed with Clustal Omega. *Arabidopsis thaliana - At, Saccharomyces cerevisiae – Sc, Drosophila melanogaster - Dm, Homo sapiens - Hs, Oxytricha trifallax - Ot, Tetrahymena thermophila - Tt, Paramecium primaurelia – PPRIM, Paramecium biaurelia* - PBI, *Paramecium sexaurelia* - PSEX, *Paramecium caudatum* - PCAUD, *Paramecium multimicronucleatum* – PMMN, *Paramecium decaurelia - PDEC, Paramecium dodecaurelia - PDODEC, Paramecium jenningsi - PJEN, Paramecium novaurelia - PNOV, Paramecium octaurelia - POCT, Paramecium quadecaurelia - PQUADEC, Paramecium tredecaurelia – PTRED, Paramecium sonenborni – PSON.*

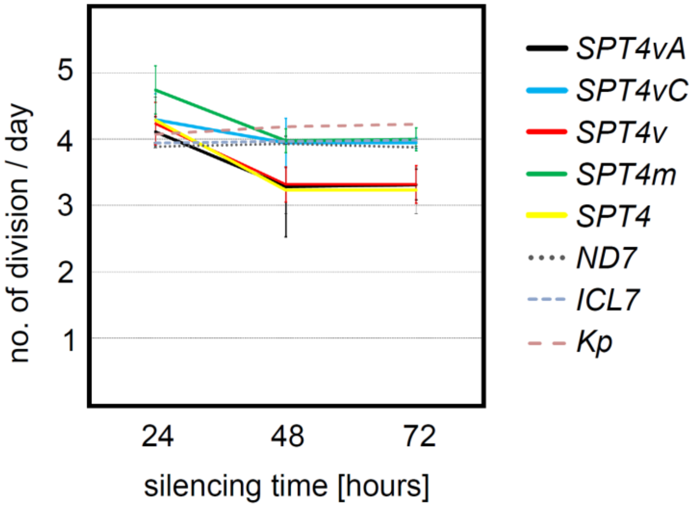

Figure S2. Silencing of *SPT4* genes during vegetative growth. The average division rate observed upon RNAi against individual *SPT4* genes (*SPT4vA, SPT4vC*), multiple *SPT4* genes (*SPT4v = SPT4vA + SPT4vC; SPT4m = SPT4mA + SPT4mB; SPT4 = SPT4mA* + *SPT4mB* + *SPT4vA*) and control non-essential genes ND7 and ICL7 or without silencing (Kp) is shown. The data summarizes results obtained for more than 18 cell lines. Standard deviation is shown with vertical bars.

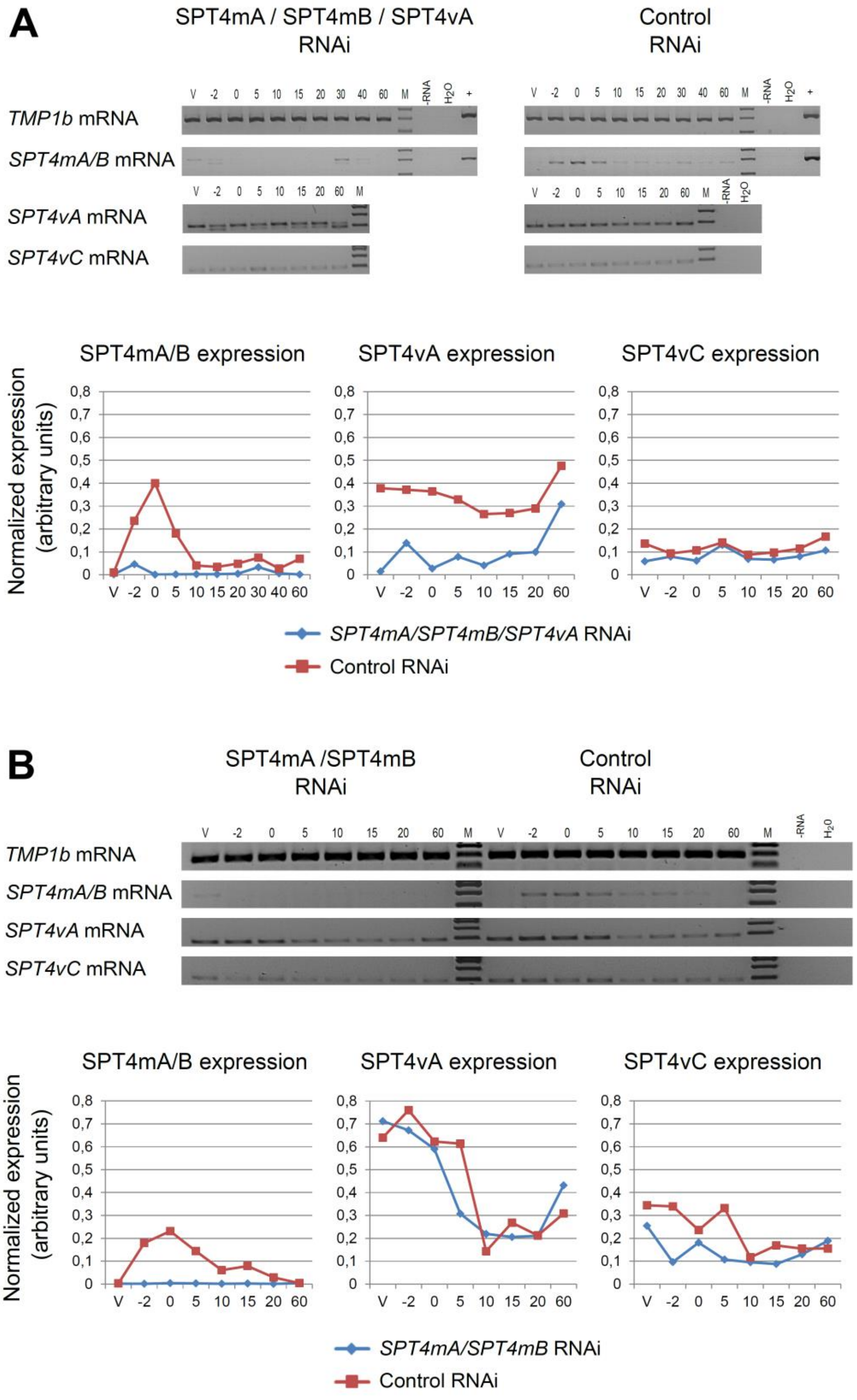

Figure S3. RT-PCR-based expression profiles of different *SPT4* genes upon *SPT4*-RNAi and *SPT4m*-RNAi during autogamy. A. Results for SPT4-RNAi silencing. The constitutively expressed unrelated gene *TMP1b* encoding a trichocyst matrix protein

was used for normalization. Detailed description of time-points used in the analysis is given in **Figure S4**. The two bands present in some time-courses (panel A, Spt4Va mRNA) represent remnants of feeding vectors containing an intron sequence (upper band) and the cDNA of a particular gene without the intron sequence (band below). Negative controls marked as "-RNA" were prepared using water instead of RNA, while controls marked as "H_2O" contain water instead of reverse transcriptase. For practical reasons these controls are shown for a single time point for each reaction, using material obtained from the Control-RNAi experiments. Positive controls marked as “+” used genomic DNA for final amplification. The charts demonstrating gene expression profiles present values acquired by analysis of the intensity of the cDNA bands with the ImageJ software. **B.** Results for *SPT4m*-RNAi. Due to the high nucleotide similarity between *SPT4mA* and *SPT4mB*, both genes were identified using the same pair of primers.

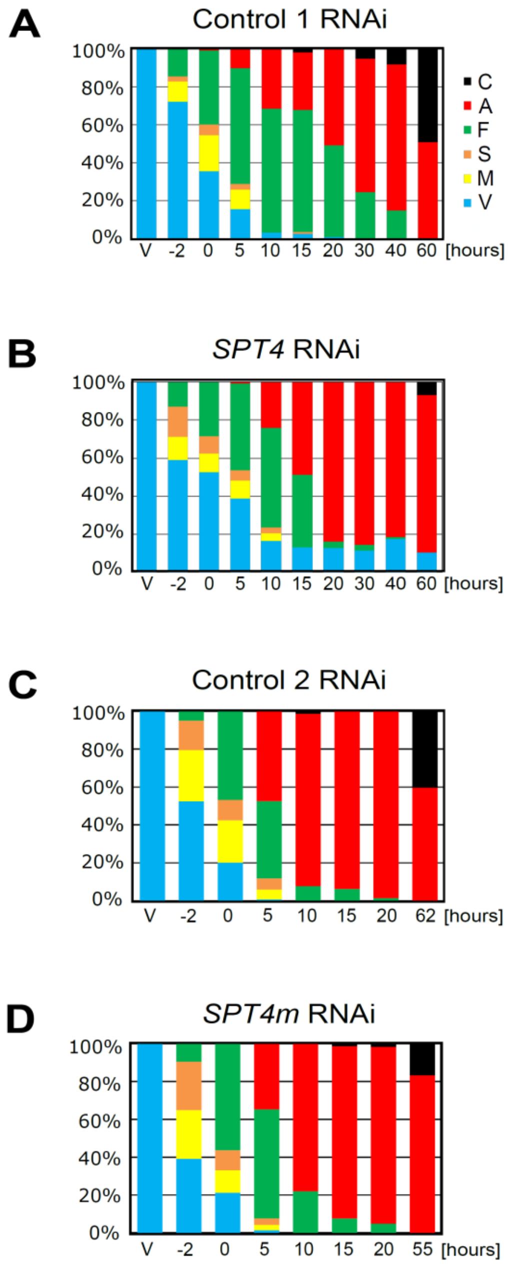

Figure S4. Autogamy time-courses of *Paramecium* cultures silenced for *SPT4* genes or with control RNAi. Histograms show the progression of autogamy in strain 51mt7 Δ*A*. As a control we used cells silenced for the unrelated *ICL7* gene. For each time-point (V: vegetative culture; -2: meiosis and early MAC fragmentation; 0: around 50% of cells with fragmented MAC; 5 to 60: 5 to 60 hours following time 0, respectively), cells were stained with DAPI to visualize nuclei. V: vegetative parental MAC; M: meiosis; S: skein formation; F: fragmented old MAC but no detectable developing new MACs; A: fragmented old MAC + 2 visible anlagen, C: post-karyonidal cells. **A.** Control 1 RNAi. **B.** SPT4-RNAi (SPT4mA, SPT4mB and SPT4vA triple silencing). **C.** Control 2 RNAi. **D.** SPT4m-RNAi (SPT4mA and SPT4mB double silencing).

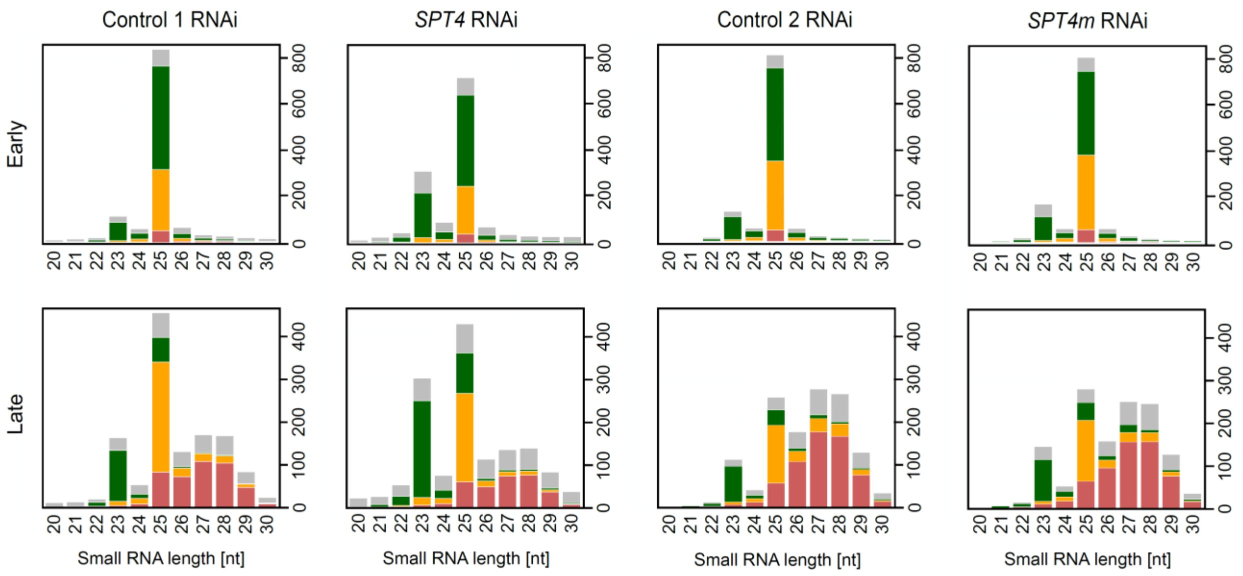

Figure S5. Results of mapping of sRNAseq reads to reference genomes. Histograms show normalized number of sequence reads corresponding to RNAs varying from 20 to 30 nt obtained at early (T0) and late (T15) time points mapped to reference genomes: MAC, MIC-limited and IES sequences.

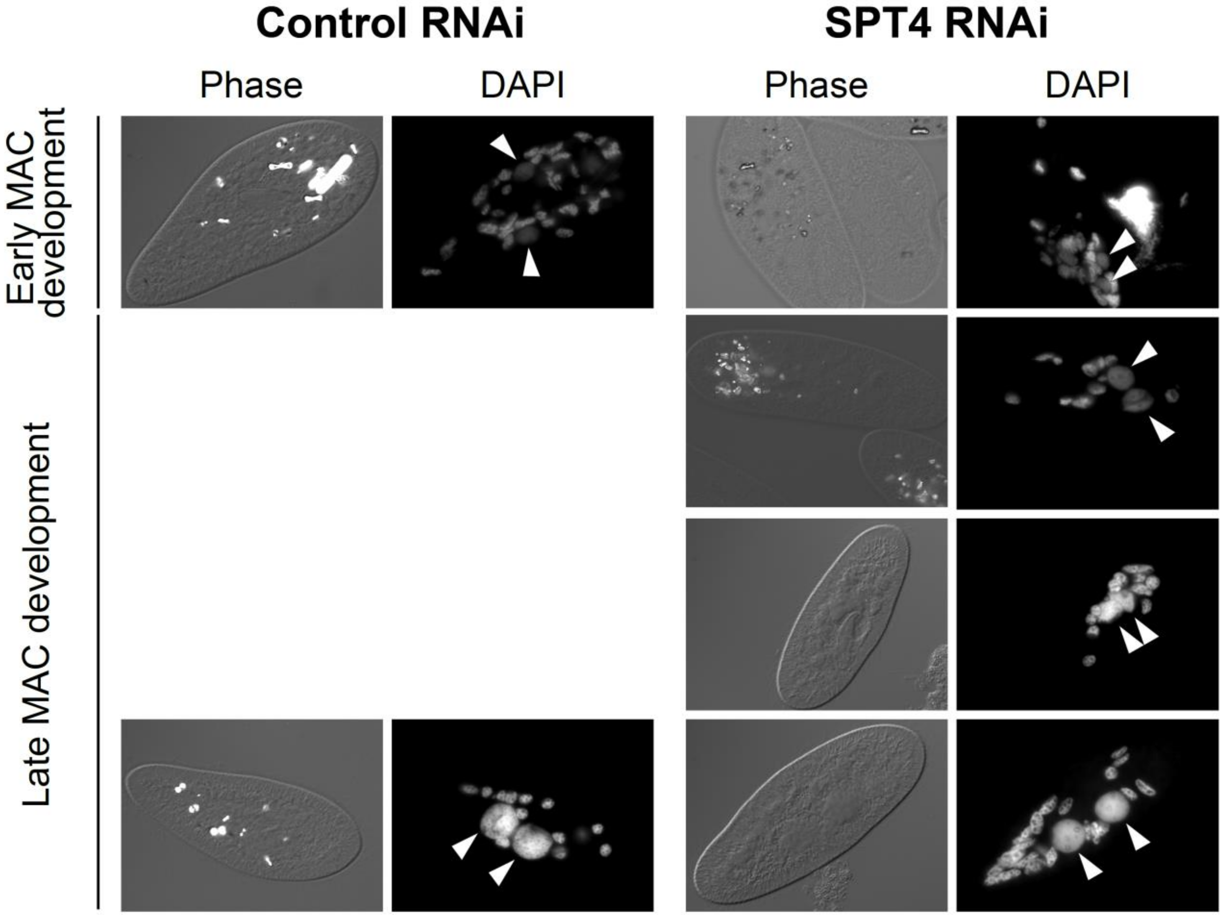

Figure S6. DNA amplification in new MACs in *SPT4*-RNAi. White arrowheads mark new developing MACs.

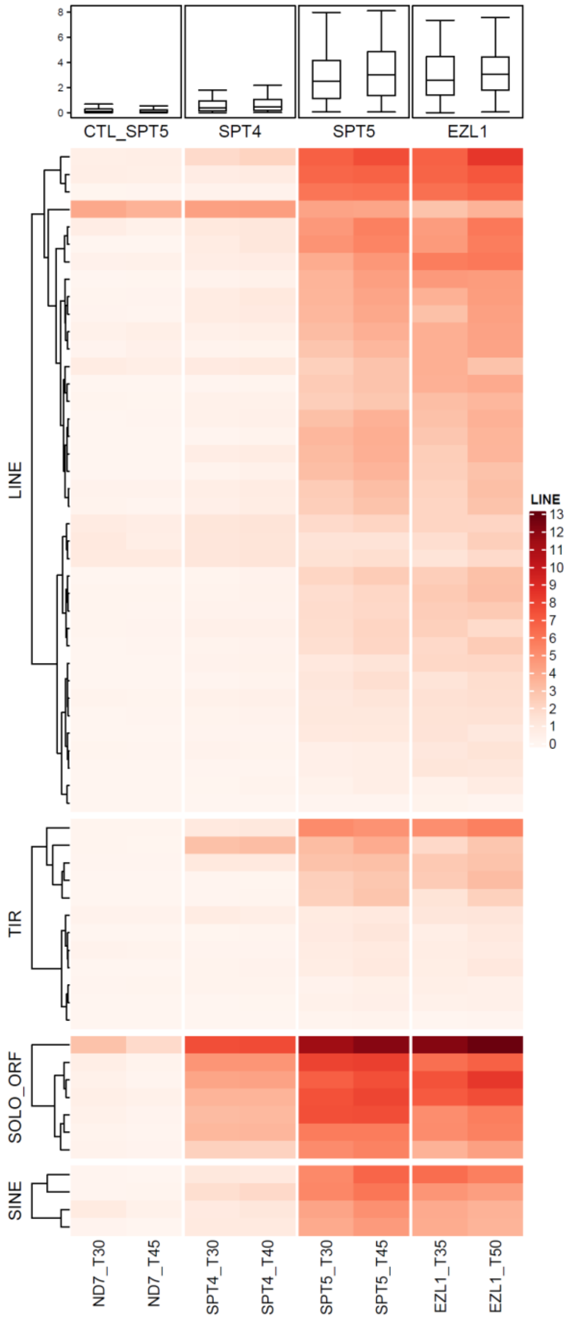

Figure S7. Heatmap of RNA expression levels at late time points during development upon silencing of control gene (*ND7*), *SPT4, SPT5m* or *EZL1* (48). Each row represents a different TE consensus sequence, and the TE consensuses are ordered by hierarchical clustering within each TE family (LINE, TIR, Solo-ORF, SINE). The boxplots above the heatmaps show the coverage (RPKM log2) for all TE consensuses.

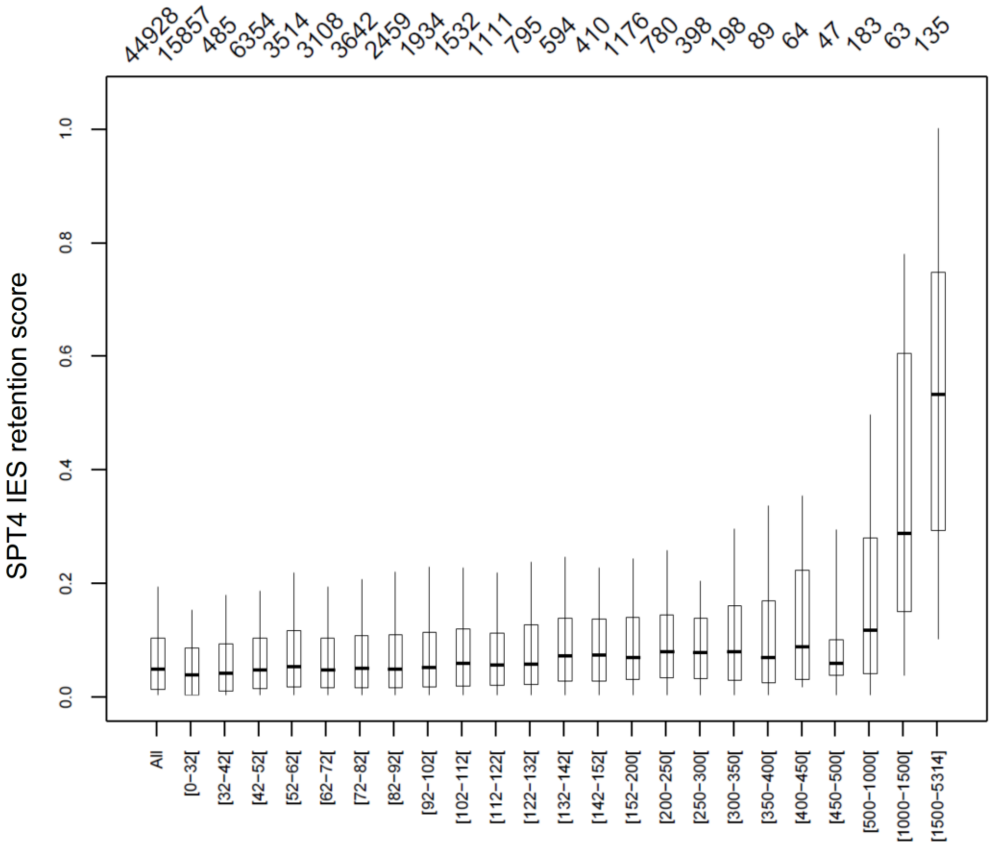

Figure S8. Relation between IES size and SPT4 IES retention score. The box plot shows the SPT4 IES retention score distribution for each group of IESs corresponding to a peak in the periodic IES size distribution (24). The median retention score (horizontal line inside the box) and the first (top of box) and third (bottom of box) quartiles as well as the 10th and 90th centiles (thin vertical line) are shown.

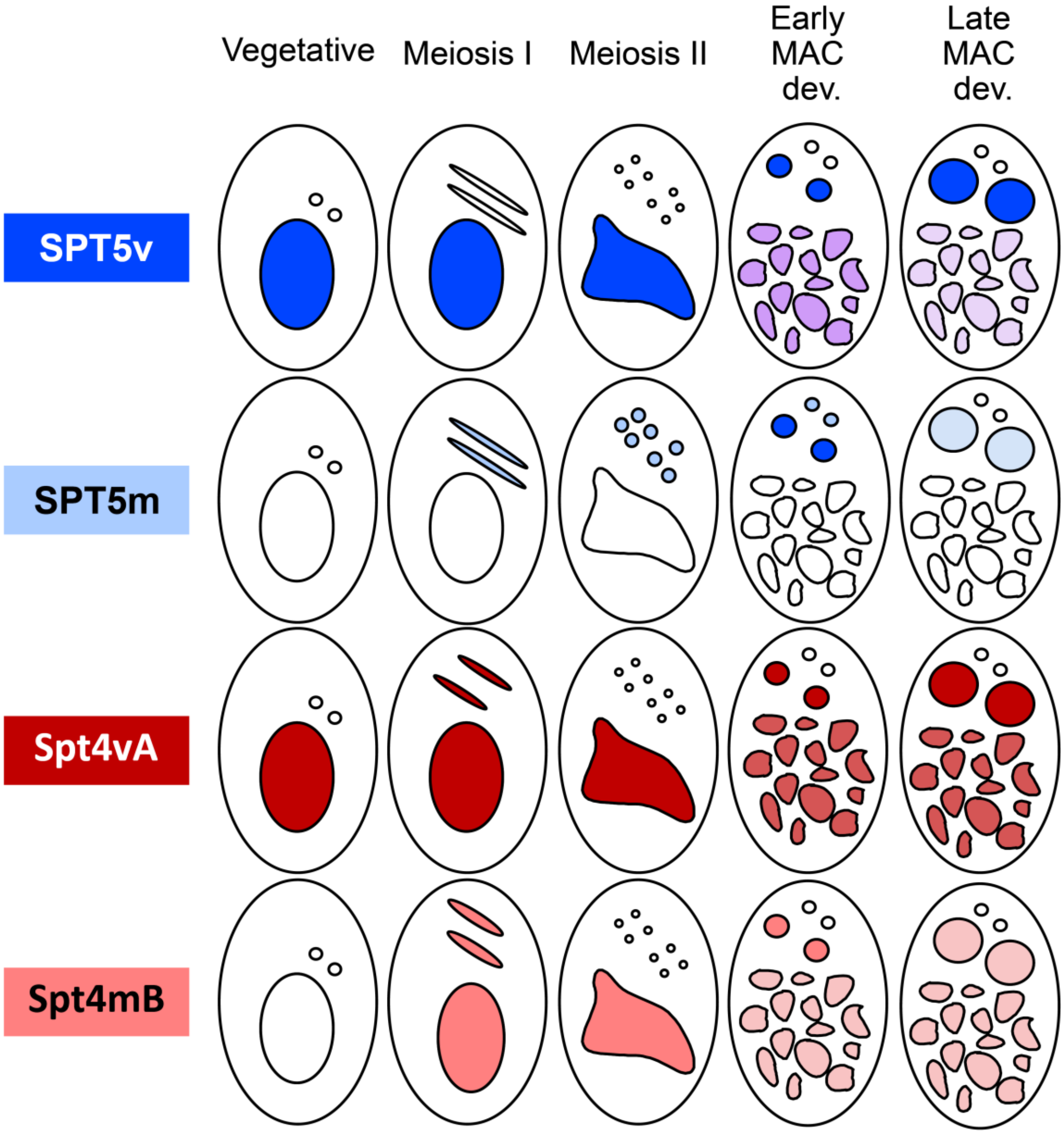

Figure S9. Scheme showing localization of Spt5 and Spt4 homologs during vegetative growth and different phases of the sexual cycle.

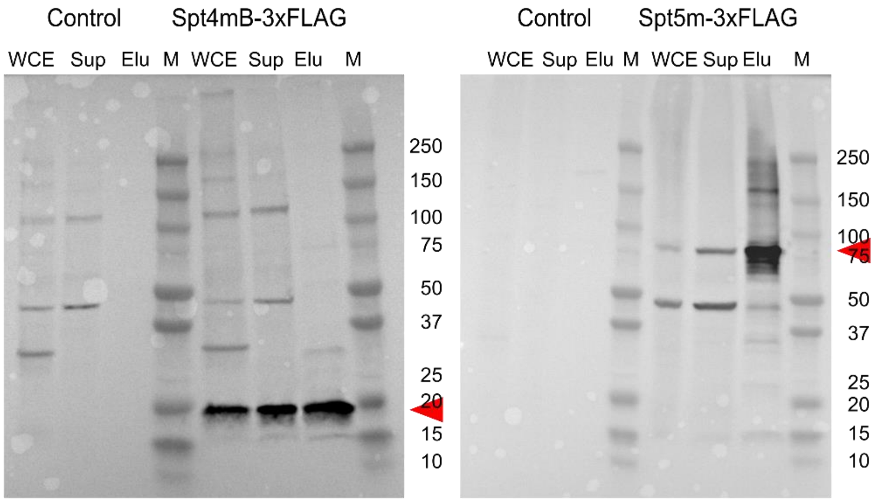

Figure S10. Identification of transgene products by Western blot analysis. (A) Spt4mB-3xFLAG, indicated by the red arrowhead, was identified specifically in transformed cells and undetected in a control culture. The signal is detected slightly below the 20-kDa protein marker band, although the predicted molecular weight is ~13 kDa. **(B)** The signal of Spt5m-3xFLAG, indicated by the red arrowhead, is also observable exclusively in material obtained from the transformed cells. Even though its predicted molecular weight is ~70 kDa, its migration overlays with the 75-kDa protein standard band. The shifts in the migration of both proteins may be related to their affinity for nucleic acids (70). In both blots abbreviations stand as follows: WCE – whole cell extract, Sup – supernatant, Elu – eluate from the Anti-Flag M2 affinity gel, M – Precision Plus Protein Standards (Bio-Rad).

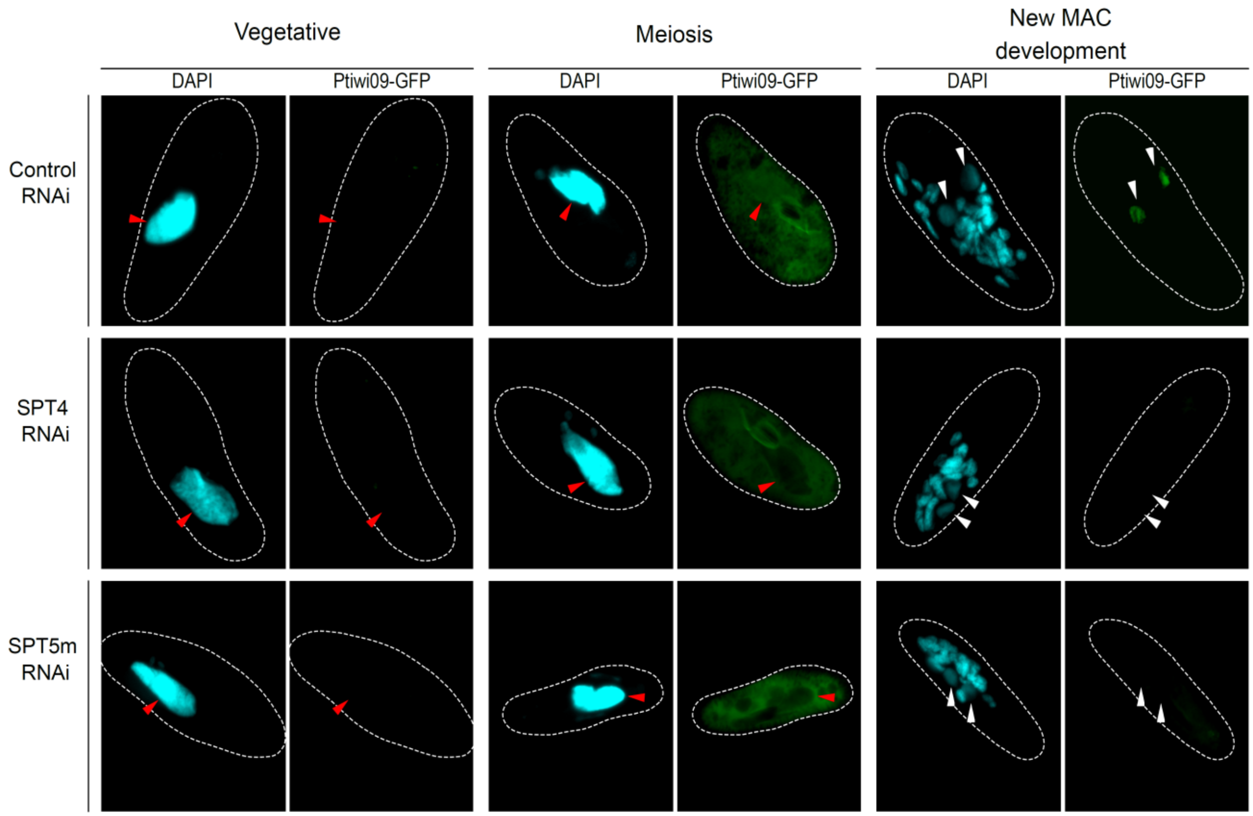

Figure S11. Localization of Ptiwi09-GFP upon silencing of *SPT4* and *SPT5m*. Ptiw09 is expressed during meiosis and localizes to the maternal MAC and cytoplasm in the control experiment but is visible only in the cytoplasm after diminution of Spt4 or Spt5m. During new MAC development Ptiwi09-GFP is present in the new MACs in the control but is not detectable in *SPT4*-RNAi and in *SPT5m*-RNAi. The contours of cells are presented by dashed white lines, red arrowheads point to maternal MACs while white arrowheads mark new developing MACs.

Supplementary Tables

Sample description	Sequence type	File name(s)	ENA accession
P. tetraurelia DNA sequencing after SPT4 depletion	DNA	PTET_SPT4_RNAi_DO_ABV_5_R1.fastq.bz2 PTET_SPT4_RNAi_DO_ABV_5_R2.fastq.bz2	ERS6269357
P. tetraurelia DNA sequencing after SPT4mAB depletion	DNA	PTET_SPT4m-AB_RNAi_S8_R1_001.fastq.bz2 PTET_SPT4m-AB_RNAi_S8_R2_001.fastq.bz2	ERS6269358
P. tetraurelia sRNA sequencing after ICL7 depletion : T0	sRNA	PTET_ICL_RNAi_sRNA_T0_S10_R1_001.fastq.bz2	ERS6269359
P. tetraurelia sRNA sequencing after ICL7 depletion : T15	sRNA	PTET_ICL_RNAi_sRNA_T15_S11_R1_001.fastq.bz2	ERS6269360
P. tetraurelia sRNA sequencing after SPT4mAB depletion : T0	sRNA	PTET_SPT4m-AB_RNAi_sRNA_T0_S12_R1_001.fastq.bz2	ERS6269361
P. tetraurelia sRNA sequencing after SPT4mAB depletion : T15	sRNA	PTET_SPT4m-AB_RNAi_sRNA_T15_S13_R1_001.fastq.bz2	ERS6269362
P. tetraurelia sRNA sequencing after SPT4 depletion : T0	sRNA	SPT4_sRNA_DO_E_T0_L001_R1_001.fastq.bz2	ERS6269363
P. tetraurelia sRNA sequencing after SPT4 depletion : T15	sRNA	SPT4_sRNA_DO_E_T15_L001_R1_001.fastq.bz2	ERS6269364
P. tetraurelia sRNA sequencing klebsiella control experiment : T0	sRNA	SPT4_sRNA_DO_K_T0_L001_R1_001.fastq.bz2	ERS6269365
P. tetraurelia sRNA sequencing klebsiella control experiment : T15	sRNA	SPT4_sRNA_DO_K_T15_L001_R1_001.fastq.bz2	ERS6269366
P. tetraurelia mRNA sequencing after SPT4 depletion : T30	mRNA	PTET_mRNA_SPT4_RNAi_T30_JkN_S128_R1_001.fastq.bz2 PTET_mRNA_SPT4_RNAi_T30_JkN_S128_R2_001.fastq.bz2	ERS10166352
P. tetraurelia mRNA sequencing after SPT4 depletion : T40	mRNA	PTET_mRNA_SPT4_RNAi_T40_JkN_S129_R1_001.fastq.bz2 PTET_mRNA_SPT4_RNAi_T40_JkN_S129_R2_001.fastq.bz2	ERS10166353
P. tetraurelia mRNA sequencing after SPT5 depletion : T30	mRNA	PTET_mRNA_SPT5_RNAi_T30_JkN_S124_R1_001.fastq.bz2 PTET_mRNA_SPT5_RNAi_T30_JkN_S124_R2_001.fastq.bz2	ERS10166354
P. tetraurelia mRNA sequencing after SPT5 depletion : T45	mRNA	PTET_mRNA_SPT5_RNAi_T45_JkN_S125_R1_001.fastq.bz2 PTET_mRNA_SPT5_RNAi_T45_JkN_S125_R2_001.fastq.bz2	ERS10166355
P. tetraurelia mRNA sequencing after ND7 depletion : T30	mRNA	PTET_mRNA_ND7_RNAi_T30_JkN_S126_R1_001.fastq.bz2 PTET_mRNA_ND7_RNAi_T30_JkN_S126_R2_001.fastq.bz2	ERS10166356
P. tetraurelia mRNA sequencing after ND7 depletion : T45	mRNA	PTET_mRNA_ND7_RNAi_T45_JkN_S127_R1_001.fastq.bz2 PTET_mRNA_ND7_RNAi_T45_JkN_S127_R2_001.fastq.bz2	ERS10166357

Table S1. Individual sequencing data sets.

		Number of peptides matching particular protein						
**Sample no.**	**Bait**	**Spt5m**	**Spt5v**	**Spt4mA/B**	**Spt4vA**	**Spt4vC**	**Ptiwi01/09**	**Experiment details**
A05.	Spt5m-3xFLAG	51	0	4	2	0	9	elution with competitive peptide
A06.	Spt5m-3xFLAG	44	0	2	0	0	0	elution with competitive peptide
A07.	Spt5m-3xFLAG	30	0	2	0	0	0	elution with competitive peptide
A09.	Control	0	0	0	0	0	0	elution with competitive peptide
A10.	Control	0	0	0	0	0	0	elution with competitive peptide
A11.	Control	0	0	0	0	0	0	elution with competitive peptide
012.	Spt5m-3xFLAG	96	0	1	1	0	0	elution with glycine
013.	Spt5m-3xFLAG	60	0	1	1	0	3	additional wash with neutral IgG; elution with glycine
020.	Spt5m-3xFLAG	161	0	2	0	0	9	elution with glycine
021.	Spt5m-3xFLAG	221	0	2	1	0	9	elution with glycine
017.	Control	0	0	0	0	0	0	elution with glycine
018.	Control	0	0	0	0	0	0	elution with glycine
019.	Control	0	0	0	0	0	0	elution with glycine
014.	Spt4mB-3xFLAG	6	19	227	0	0	0	elution with competitive peptide
015.	Spt4mB-3xFLAG	10	21	62	0	0	1	elution with glycine
045.	Spt4mB-3xFLAG	9	141	56	0	0	1	direct trypsin digestion of washed resin
046.	Spt4mB-3xFLAG	15	80	24	0	0	3	direct trypsin digestion of washed resin
047.	Spt4mB-3xFLAG	8	76	22	0	0	3	direct trypsin digestion of washed resin
048.	Control	0	0	0	0	0	5	direct trypsin digestion of washed resin

Table S2. Summary of mass spectrometry experiments.
